# Targeting CXCR2 inhibits the progression of lung cancer and promotes therapeutic effect of cisplatin

**DOI:** 10.1186/s12943-021-01355-1

**Published:** 2021-04-04

**Authors:** Yuan Cheng, Fei Mo, Qingfang Li, Xuejiao Han, Houhui Shi, Siyuan Chen, Yuquan Wei, Xiawei Wei

**Affiliations:** 1 Laboratory of Aging Research and Cancer Drug Target, State Key Laboratory of Biotherapy, National Clinical Research Center for Geriatrics, West China Hospital, Sichuan University, No. 17, Block 3, Southern Renmin Road, Chengdu, Sichuan 610041 People’s Republic of China; 2 Department of Medical Oncology, First Affiliated Hospital of Kunming Medical University, Kunming, Yunnan People’s Republic of China

**Keywords:** CXCR2, SB225002, Lung cancer, Neutrophils, T cells, Cisplatin

## Abstract

**Background:**

Drug-resistance and severe side effects of chemotherapeutic agents result in unsatisfied survival of patients with lung cancer. CXCLs/CXCR2 axis plays an important role in progression of cancer including lung cancer. However, the specific anti-cancer mechanism of targeting CXCR2 remains unclear.

**Methods:**

Immunohistochemical analysis of CXCR2 was performed on the microarray of tumor tissues of clinical lung adenocarcinoma and lung squamous cell carcinoma patients. CCK8 test, TUNEL immunofluorescence staining, PI-Annexin V staining, β-galactosidase staining, and Western blot were used to verify the role of CXCR2 in vitro. Animal models of tail vein and subcutaneous injection were applied to investigate the therapeutic role of targeting CXCR2. Flow cytometry, qRT-PCR, enzyme-linked immunosorbent assay (ELISA), and immunohistochemistry analysis were performed for further mechanistic investigation.

**Results:**

The expression of CXCR2 was elevated in both human lung cancer stroma and tumor cells, which was associated with patients’ prognosis. Inhibition of CXCR2 promoted apoptosis, senescence, epithelial-to-mesenchymal transition (EMT), and anti-proliferation of lung cancer cells. In vivo study showed that tumor-associated neutrophils (TANs) were significantly infiltrate into tumor tissues of mouse model, with up-regulated CXCLs/CXCR2 signaling and suppressive molecules, including Arg-1 and TGF-β. SB225002, a selective inhibitor of CXCR2 showed promising therapeutic effect, and significantly reduced infiltration of neutrophils and enhanced anti-tumor T cell activity via promoting CD8^+^ T cell activation. Meanwhile, blockade of CXCR2 could enhance therapeutic effect of cisplatin via regulation of neutrophils infiltration.

**Conclusions:**

Our finds verify the therapeutic effects of targeting CXCR2 in lung cancer and uncover the potential mechanism for the increased sensitivity to chemotherapeutic agents by antagonists of CXCR2.

**Supplementary Information:**

The online version contains supplementary material available at 10.1186/s12943-021-01355-1.

## Introduction

Lung cancer is the leading course of malignancy-related mortality worldwide. More importantly, it has surpassed breast cancer and become the leading course of cancer death in more developed areas. Because of air pollution, lung cancer incidence of Chinese women is higher than that of some European countries women despite a lower prevalence of smoking [1]. Data has shown a fast-growing prevalence of lung cancer in non-smoking women between 30 and 50 years old [2]. Among all lung cancer cases, non-small cell lung cancer (NSCLC) accounts for more than 85% [3]. Despite the efforts made on the treatment strategies, survival of NSCLC patients remains unsatisfied, with a 5-year-survival around 17% [4]. Drug-resistance developed during treatment is one of the reasons causing low survival rate in lung cancer patients [5]. Platinum-based regimens are first-line treatments for lung cancer chemotherapy. Currently, the platinum compounds used in NSCLC patients are cisplatin and carboplatin. Their antitumor activity are mainly based on the formation of platinum-DNA adducts, which induces DNA damage and cancer cell apoptosis. Surgical resection either following (neoadjuvant) or followed by (adjuvant) platinum agents is standard therapy for a resectable NSCLC patients [6]. However, drug-resistance and obvious side effect of platinum-based drugs make combined therapy and targeted therapy promising strategies. During the latest decades, small molecular targeted therapies, such as receptor tyrosine kinase inhibitors (TKIs) and immune checkpoint inhibitors, have provided new approach to prove the treatment of NSCLC patients [7, 8]. The development of immune checkpoint blockade (ICB) therapy has revolutionized the treatment of advanced NSCLC. For now, several immune checkpoint inhibitors have been approved by the US Food and Drug Administration (FDA) for the treatment of NSCLC, including the PD-1 inhibitor Nivolumab, Pembrolizumab and, PD-L1 inhibitor Atezolizumab and Durvalumab [9]. However, despite the application of these new treatment strategies, multiple immune resistance mechanisms within tumor microenvironment result in less satisfactory therapeutic effect.

Tumor microenvironment is consist of various non-malignant cells like leukocytes, fibroblasts and endothelial cells, and non-cellular component, such as cytokines and chemokines. Macrophages and neutrophils are two kinds of myeloid cells abundant in tumor microenvironment. Tumor-associated immunosuppressive myeloid cells have close relationship with poor prognosis and ineffective treatment [10, 11]. Tumor-associated neutrophils (TANs) are polarized by various factors in tumor microenvironment, such as hypoxia and cytokines. For example, TANs are polarized to N2 type after exposed to TGF-β, which is a type of pro-tumor TANs, and IFN-γ is capable to transform TANs into N1 type, which shows snit-tumor effect [12, 13]. Studies have shown pro-tumor TANs (N2 type) usually hold a leading position in progressive cancer and have been associated with aggressive types of cancer and worse clinical outcomes [14]. Recent studies has proven targeting tumor-associated immunosuppressive myeloid cells enhanced antitumor activity against lung cancer [15].

Since the first CXC chemokine, IL-8, was identified in 1980s, there have been many studies on IL-8 and its receptors [16]. Two IL-8 receptors were found: IL-8 RA and IL-8 RB, also known as CXCR1 and CXCR2 [17]. CXCR2 shares 77% sequence homology with CXCR1, and they both bind to IL-8 with similar affinity (Kd of approximately 4 nM) [18, 19]. However, CXCR1 only binds to CXCL6 and CXCL8 in human and an ortholog of CXCL8 is missing in mouse, instead, CXCR1 is activated by CXCL1 and CXCL6, which indicates CXCR2 interacts with more ELR^+^ chemokines with higher affinity and plays a more vital role in chemotaxis of cells [20, 21]. CXCR2 is a typical G-protein-coupled receptor, which is responsible for human CXC chemokines including CXCL1, CXCL2, CXCL3, CXCL5, CXCL6, CXCL7 and CXCL8. CXCR2 with its ligands shows powerful chemotaxis of neutrophils or myeloid-derived suppressor cell (MDSC) and is related to tumor angiogenesis, progression and chemoresistance [22–26]. The expression of CXCR2 on myeloid cells from tumor bearing objectives is much higher than that of healthy objectives, which causes the migration of myelocytes to the tumor [27, 28]. In lung adenocarcinoma, CXCR2 is a poor prognostic marker and its expression is associated with tumor invasion and metastasis [29]. Previous studies have already proved that high level of CXCR2 on lung cancer cells is associated with smoking and poor prognosis in clinical patients [29]. Inhibition of CXCR2 and its ligand CXCL8 significantly inhibits proliferation and migration of lung cancer cells and decreases angiogenesis [29–31]. Meanwhile, CXCLs/CXCR2 axis is thought to have close relationship with tumor drug-resistance [32–34]. The expressions of CXCR2 and its ligands are elevated during oxaliplatin treatment in prostate cancer [35]. However, the specific mechanism underlying the combined therapy of targeting CXCR2 and chemotherapeutic drugs is unclear. Radiation therapy is another important treatment for NSCLC as a main treatment or an adjuvant therapy. The alteration of CXCLs/CXCR2 axis has also been observed after radiotherapy with recruitment and activation of neutrophils [36]. The finds of these studies indicated CXCLs/CXCR2 axis played a potential role during chemotherapy or radiotherapy for NSCLC.

In this study, we used tumor tissue microarray of lung cancer patients and established animal models to investigate the role CXCLs/CXCR2 signaling played in lung cancer.

## Materials and methods

### Patient specimens and tissue microarrays

The immunohistochemistry of a total of 93 human lung adenocarcinoma and 90 lung squamous cell carcinoma tissues was done by Shanghai Outdo Biotech Company, Shanghai, China. The anti-CXCR2 antibody was purchased from Abcam (ab14935). Intensity of immunohistochemical staining of CXCR2 in tumor tissue was scored by two independent pathologists according to semi-quantitative immunoreactivity scoring (IRS) system. The intensity was scored as 0 (no immunostaining), 1 (weak immunostaining), 2 (moderate immunostaining) and 3 (strong immunostaining). The percentage of positive cells in tumor stroma was documented as 0 (none), 1 (< 10%), 2 (10–50%), 3 (51–80%) and 4 (> 80%). The intensity of immunostaining score and the percentage of immunoreactive cells were multiplied to get IRS ranging from 0 to 12. The optimum cut-off values were 5.0 for adenocarcinoma and 5.5 for squamous cell carcinoma which were Youden indexes from receiver operating characteristic (ROC) curves based on the association with the patients’ overall survival. Kaplan-Meier survival analysis was used to determine the relationship between CXCR2 expression and patients’ survival.

### Cell proliferation and apoptosis assay

Cell proliferation was evaluated by Cell Counting Kit-8(CCK-8; Dojindo, Kumamoto, Japan). Briefly, 5 × 10^3^ cells of LL2 cells were plated in 96 well plates and were incubated with either 0.1% (v/v) DMSO (control) or various concentrations of SB225002 (S7651, Selleck, a potent, and selective CXCR2 antagonist with > 150-fold selectivity over CXCR1) for 24 h, 48 h and 72 h at 37 °C. After treatment, cells were incubated in 10% CCK-8 reagent for another 2 h. The OD value was measured at 450 nm with a microplate reader from Bio-Rad (Microplate reader 3550-UV). For apoptosis assay, 5 × 10^5^ cells were plated in 6-well plates and incubated with culture medium in the presence of DMSO or various concentrations of SB225002. After 24 h, cells were harvested and washed twice with cold PBS. Those cells were stained with Annexin V/propidium iodide (PI) (BD Biosciences) and examined by NovoCyte Flow Cytometer (ACEA Biosciences) and data was analyzed by NovoExpress® software (1.3.0, ACEA Biosciences). According to TdT-mediated dUTP Nick-End Labeling (TUNEL) assay kit (Promega), treated cells were plated in 24-well plates on coverslips and stained with TUNEL reagent and DAPI. The coverslips were then moved on slides after washed with PBS. TUNEL staining was analyzed with a fluorescence microscopy (Eclipse 80i; Nikon Co., Tokyo, Japan).

### Western blot analysis

CXCL2 was chosen for activation of CXCR2 in this experiment because of its expression in LL2 and H460 cells and avoidance of trans-activation of CXCR1. The inhibitors of SB225002 or CXCL2 (C096 & C096, Novoprotein) was added to the LL2 or H460 cells culture medium for 24 h. After the incubation, the tumor cells were harvested and immediately lysed with RIPA lysis buffer (Beyotime Institute of Biotechnology) containing proteinase inhibitor cocktail (Sigma-Aldrich). The total proteins were collected and the concentrations were determined by BCA protein assay (Pierce, Thermo Fisher Scientific). Equal amounts of proteins were electrophoresed and separated by SDS-PAGE gels, transferred onto Millipore PVDF membranes and blocked with 5% BSA solution. Then, the membranes were incubated at 4 °C overnight with primary antibodies to MAPK pathway-associated proteins (p38/p-p38, ERK/p-ERK, and JNK/p-JNK), senescence-associated proteins (p16 and p21), EMT-associated proteins (E-cadherin, Vimentin, and Snail), GAPDH, and β-actin. The anti-p38 (8690), anti-p-p38 (9211), anti-ERK (4695), anti-p-ERK (4370), anti-JNK (9252), anti-p-JNK (4668) were purchased from Cell Signaling Technology. The anti-p16 (51243), anti-p21 (109199), anti-E-cadherin (76055), anti-Vimentin (92547), anti-Snail (53519) were obtained from Abcam. The anti-GAPDH (R1108–1) and anti-β-actin (R1207–1) were purchased from HuaBio. Antibodies were detected using HRP-conjugated secondary antibody (Abcam) by an enhanced chemiluminescence detection kit (Immobilon™ Western Chemiluminescent HRP Substrate). The blots were tested for β-actin or GAPDH to confirm equal protein loading.

### Animals

Female C57BL/6 wild type mice were purchased from Vital River (6–8 weeks old, weighting 18–22 g, Beijing, China). The mice were housed and maintained under specific-pathogen-free (SPF) conditions in an animal facility. All of the animal experiments were performed according to the guidelines of the Institutional Animal Care and Use Committee of Sichuan University (Chengdu, Sichuan, China) and protocols were approved by the Institutional Animal Care and Use Committee of Sichuan University (Chengdu, Sichuan, China). No blinding experimental method was used in this study.

### Tumor challenge and treatment experiments

In vivo experiment, 6–7 female C57BL/6 mice were used in each group. The murine cancer cell lines for lung cancer, Lewis lung cancer (LLC, LL2) cell line was purchased from ATCC and maintained at DMEM medium supplemented with 10% fetal bovine serum (FBS) and penicillin. CXCR2 inhibitor SB225002 was dissolved at 1% DMSO, 20% polyethylene glycol 400, 5% tween 80, and 74% ddH_2_O. Cisplatin (15663–27-1) was purchased from MedChemExpress. Based on our preliminary experimental results and other similar researches [37, 38], SB225002 was administered at 10 mg/kg by intraperitoneal (i.p.) injection every day and cisplatin was administered at 2.5 mg/kg, i.p., once a week. In combination therapy, treatment of cisplatin was initiated 3 days after first injection of SB225002. Control groups received solvent (1% DMSO, 20% PEG 400, and 5% Tween 80). On day 0, LL2 cells were collected and re-suspended in serum- and penicillin-free medium. For subcutaneous tumor model, 100 μl cell suspension containing 1 × 10^6 was injected subcutaneously in the right flank and treatment was started when the tumors were palpable. Mice were sacrificed on day 25–28. For orthotopic lung cancer model, 100 μl cell suspension containing 5 × 10^5 cells was injected intravenously through tail vein and treatment was started on day 3 ending on day 21. All mice were randomly assigned to cohorts. Mice were sacrificed on day 23–25.

### Flow cytometry analysis

The lungs containing tumor nodules were collected after mice were sacrificed. Mouse lung tissues were dissected and cut into small pieces and were digested into single-cell suspension by 1 mg/mL collagenase Type I in RPMI 1640 basic medium for 2 h in 37 °C. The red blood cell lysis buffer (154 mM NH_4_Cl, 10 mM KHCO_3_, 0.1 mM EDTA, pH 7.4) was then added to the single-cell suspension to lyse red blood cells (RBC). Digested cells were washed for three times and resuspended by phosphate-buffered solution (PBS). Cells were counted, dispersed in PBS at 1 × 10^6^ cells/mL and stained with 1 μl fluorescence-conjugated antibodies (BD Biosciences, 1:100) for 30 min in 100 μl PBS at 4 °C. Cells were then washed two times before flow cytometry analysis. PerCP-Cy5.5-labelled rat anti-mouse CD45, FITC-labelled rat anti-mouse CD11b, AP -labelled rat anti-mouse Ly6C, PE-labelled rat anti-mouse Ly6G, PerCP-Cy5.5-labelled rat anti-mouse CD3, FITC-labelled rat anti-mouse CD4, APC-labelled rat anti-mouse CD8, PE-labelled rat anti-mouse CD69 were used. Data acquisition was performed on NovoCyte Flow Cytometer (ACEA Biosciences, Inc., San Diego, CA, USA) and data was analyzed by NovoExpress® software (1.3.0, ACEA Biosciences, Inc., San Diego, CA, USA, 2018).

### Quantitative real-time PCR

Reverse transcription polymerase chain reaction (RT-PCR) was used to measure mRNA transcription levels of chemokines CXCL1, CXCL2, CXCL3, CXCL5, CXCL6, CXCL7 and MIF, and chemokine receptors CXCR1 and CXCR2 of human and mouse, CXCL8 of human, EMT associated markers E-cadherin, N-cadherin, Vimentin, and Snail of human and mouse, senescence associated markers p16 and p21 of human and mouse, and cytokines Arg-1, TNF-α, TGF-β, and IFN-γ of mouse. Tumor samples or cell samples were disintegrated and homogenised mechanically using the Tissue Lyser (Qiagen) in RNase-free tubes. Total RNA was extracted using RNA simple Total RNA Kit (TIANGEN, Beijing, China) and then isolated RNA was reverse-transcribed into cDNA using Takara kit (Dalian, China). Primers used were listed in supplementary Table 1. Real-time PCR was performed with SYBR Green supermix (Bio-Rad Laboratories, Hercules, CA, U.S.) using a two-step PCR reaction procedure. Expression of those genes was normalized to the expression of GAPDH. Dates were analyzed and showed in ΔΔCt or 2^-ΔΔCt^ method.

### Isolation and chemotaxis assay of neutrophils

All bone marrow cells were harvested from femur and tibia by flushing bone cavities using culture medium without FBS and filtered through 70 μm nylon mesh following RBC lysis. The cell suspension was carefully layered onto Histopaque-1077/1119 gradient solutions (Sigma) and was centrifuged at 700 g for 30 min. After centrifugation, the neutrophils were aspirated from the responding layer in accordance with the manufacturer’s instruction. The cells were then cultured in RPMI 1640 medium supplemented with 10% FBS, penicillin, and streptomycin. Supernatants of LL2 tumor cells were collected 24 h after the cells reached 70–80% density. Isolated neutrophils were seeded in upper chamber of Transwell system, and 1640 RPMI medium, tumor supernatant (TS), CXCL2 or SB225002 were added to the lower chamber. After 6 h of incubation, the number of migrated neutrophils was calculated by flow cytometry.

### T cell suppression study

Spleens were removed from 8-week female C57BL/6 wild-type mice of and placed in a 70 μm cell sieve and gently grind the spleen until no obvious tissue mass was seen. The n 4–5 ml of lymphocyte separation fluid (Dakewe, China) was added to re-suspend the tissues. The cell suspension was placed in a 15 ml centrifuge tube for gradient centrifugation at 800 g for 30 min. Lymphocytes were purified from the liquid and stained with CFSE (Invitrogen). The CFSE-labelled lymphocytes were placed in 24-well plates supplemented complete RPMI medium with 1 μg/ml anti-CD3 (R&D Systems) and 5 μg/ml anti-CD28 (R&D Systems) antibodies. Isolated neutrophils (1:1) or tumor supernatant (50%, v/v) were added to the 24-well plate. After incubation for 72 h, T cells were collected and stained with anti-CD3, anti-CD4, and anti-CD8 antibodies for flow cytometric analysis.

### Immunohistochemistry and H&E Staining

Immunohistochemistry analyses of CXCR2 expression, tumor microenvironment were done with staining with anti-mouse CXCR2 Ab (Abcam), anti-mouse Ly6G Ab (Servicebio), anti-mouse TGF-β Ab(Abcam), and anti-mouse TNF-α Ab (Abcam) using the labeled streptavidin-biotin method. Briefly, paraffin-embedded tissue sections were dewaxed with xylene and rehydrated through graded concentrations of ethanol. Endogenous peroxide was blocked with 3% H_2_O_2_ for 10 min at room temperature in the dark. Antigen retrieval was done by heating in an autoclave in 10 mM sodium citrate buffer (pH 6.0) for 3 min. Nonspecific binding sites were blocked with normal goat serum for 40 min at 37 °C followed by incubation with primary antibody overnight at 4 °C. Then the slides were incubated with the appropriate secondary antibody conjugated to HRP at 37 °C for 40 min and streptavidin-biotin complex at 37 °C for another 40 min. HRP was detected with diaminobenzidine peroxide solution and cell nuclei were gently counterstained with hematoxylin (BeyotimeInstitute of Biotechnology, Shanghai, China). After the sections were hydrated as above, the tissue sections were stained with hematoxylin and eosin (HE) for histomorphometric analysis.

### Statistical analysis

Two groups were compared with Prism software (GraphPad) using a two-tailed unpaired Student’s t-test or Dunnet’s t-test. Multiple groups were compared by using One-Way ANOVA. All data were represented as mean ± SD or mean ± SEM. Differences were considered statistically significant if *p* values < 0.05.

## Results

### CXCR2 is elevated in human lung cancer tissues and correlates with poor prognosis

**Fig. 1:**
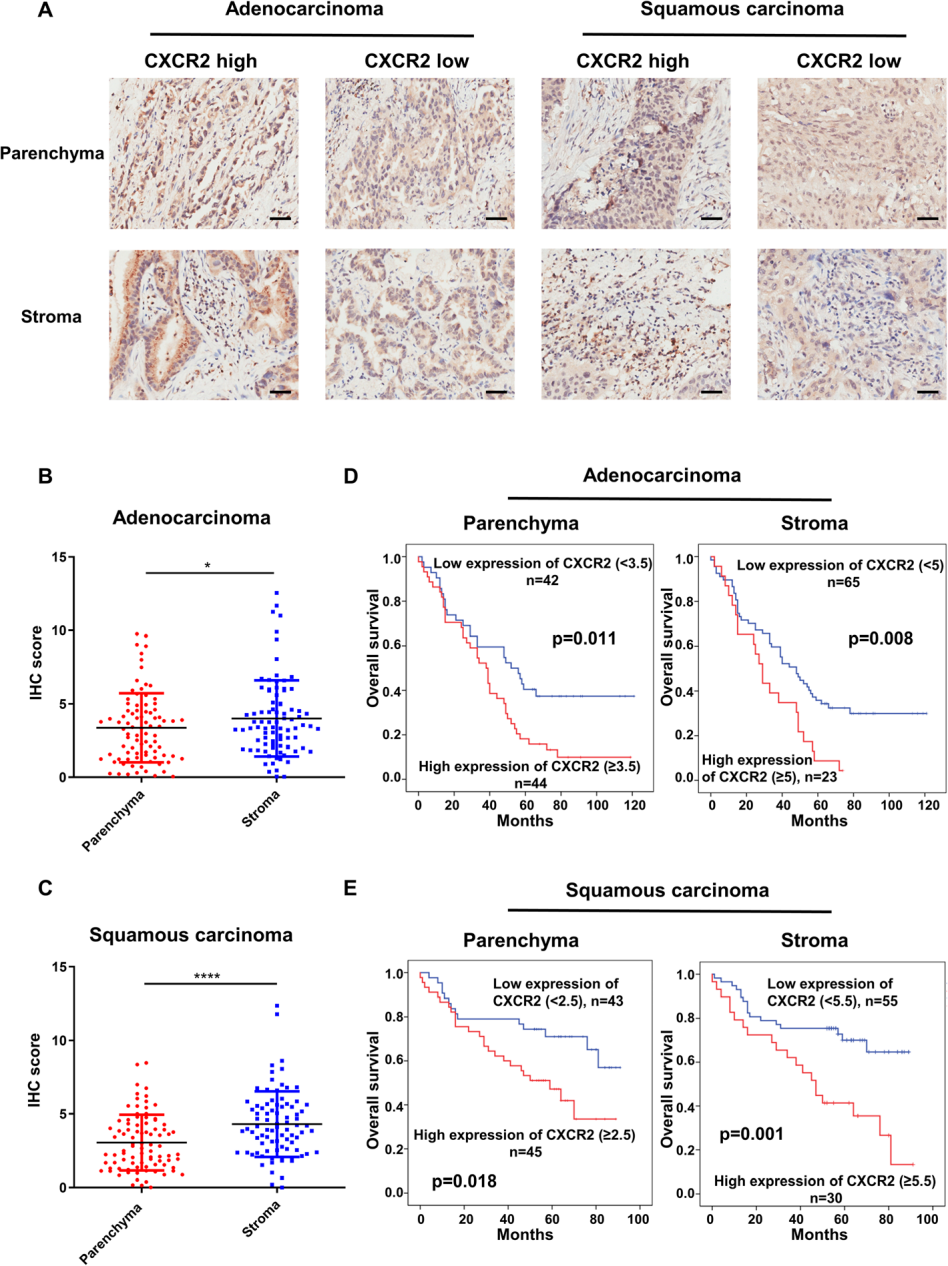
Elevated expression of CXCR2 is associated with poor prognosis of lung cancer patients. Immunohistochemical staining of CXCR2 was performed on the tumor pathological tissue microarrays of 93 patients with lung adenocarcinoma and 90 patients with lung squamous carcinoma. **a**, IHC analysis of CXCR2 expression in parenchyma and stroma of lung adenocarcinoma tissues and lung squamous cell carcinoma tissues. Scale bar, 50 μm. **b**-**c**, IHC scores of tumor cells and stroma cells of lung adenocarcinoma tissues (**b**) and lung squamous cell carcinoma tissues (**c**) (independently interpreted by two researchers, *p* = 0.046 and *p* < 0.001, respectively). The percentage of positive cells in tumor stroma was documented as 0 (none), 1 (< 10%), 2 (10–50%), 3 (51–80%) and 4 (> 80%). The intensity of positive cells was scored as 0 (no immunostaining), 1 (weak immunostaining), 2 (moderate immunostaining) and 3 (strong immunostaining). The immunostaining score and the percentage of immunoreactive cells were multiplied to get IRS ranging from 0 to 12. Data was shown as mean ± SEM. **d**-**e**, Tumor cells and stromal cells were divided into CXCR2 high-expression group and CXCR2 low-expression group according to the IHC score. The optimum cut-off values of IHC were 5.0 for adenocarcinoma and 5.5 for squamous cell carcinoma which were based on the Youden indexes from receiver operating characteristic (ROC) curves. The overall survival of lung adenocarcinoma (**d**) and lung squamous cell carcinoma patients (**e**) were compared by Kaplan-Meier survival curves and the log-rank test. IHC, immunohistochemical. *p < 0.05, **p < 0.01, ***p < 0.001, ns represents *p*>0.05

To investigate the importance of CXCR2 in human lung cancer, 90 lung squamous carcinoma and 94 lung adenocarcinoma patients’ tumor tissues were collected, and the relationship between expression of CXCR2 and lung cancer patients’ prognosis was analyzed. The baseline characteristics of the patients enrolled in this study were listed in Table 1. The IHS scoring system utilized and percentages of the weak, moderate and strong groups was presented in supplementary Fig. 1 with representative images. The result showed that CXCR2 was positive in both tumor cells and tumor stroma of most lung adenocarcinoma and squamous cell carcinoma (Fig. 1a). Based on the IHC scores of each tumor tissue, CXCR2 expression in tumor stoma was higher than that on tumor cells (Figs. 1b and c). Furthermore, patients with lung cancer were divided into CXCR2-high and CXCR2-low groups according to the Youden’s index of receiver operating characteristic (ROC) curves for prognosis of lung cancer. The data indicated that high expression of CXCR2 in human lung cancer tissues, both in stroma and parenchyma, was significantly associated with shorter survival (Figs. 1d and e). These results made CXCR2 an important negative prognostic factor in human lung cancer.

**Table 1:** Baseline characteristics of enrolled lung cancer patients

Characteristic	Adenocarcinoma		Squamous carcinoma	
	N	%	N	%
Enrolled patients^*^	94	–	90	–
Sex				
Male	51	54.3	84	93.3
Female	43	45.7	6	6.7
Age (years)	62.2 (30–84)		62.5 (8–78)	
< 60	37	39.4	29	33
> =60	57	60.6	59	67
Pathological grade				
I	9	9.6	13	14.4
II	74	78.7	72	80
III	11	11.7	5	5.6
Stage of disease (TNM)				
I	29	31.2	27	31.8
II	33	35.5	43	50.6
III	30	32.3	14	16.5
IV	1	1.1	1	1.2
Tumor (of TNM)				
1	19	20.2	13	15.5
2	52	55.3	51	60.7
3	17	18.1	17	20.2
4	6	6.4	3	3.6
Regional Lymph Nodes (of TNM)				
x	17	18.3	15	17.4
0	39	41.9	49	57
1	16	17.2	14	16.3
2	16	17.2	8	9.3
3	5	5.4	15	17.4
Expression of CXCR2 (stroma)				
Low	67	74.4	57	66.3
High	23	25.6	29	33.7
Expression of CXCR2 (parenchyma)				
Low	42	48.8	43	48.9
High	44	51.2	45	51.1

*Patients enrolled had no distant metastasis

### CXCLs/CXCR2 axis promotes lung cancer cells proliferation and anti-apoptosis

**Fig. 2:**
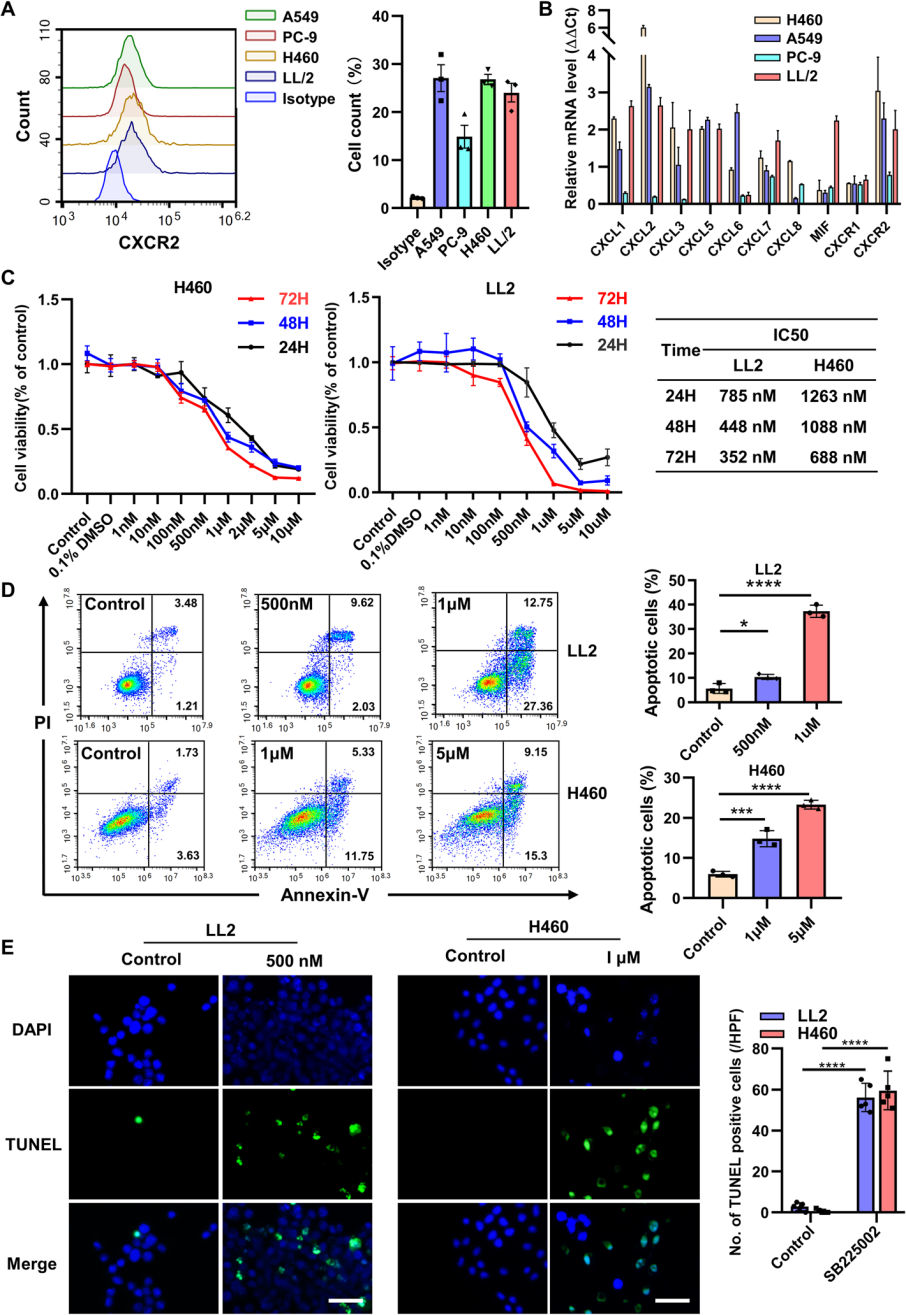
CXCLs/CXCR2 axis promotes lung cancer cells proliferation and anti-apoptosis. **a**, Cultivated LL2, A549, PC-9, H460 cells were harvested for flow cytometric analyses of CXCR2 expression on the cell surface. Data was shown as mean ± SD, *n* = 3. **b**, Total mRNA of lung cancer cells was collected for analyses of relative mRNA expression levels of CXCL1, CXCL2, CXCL3, CXCL5, CXCL7, CXCL8 (human) and MIF in lung cancer cell line. Data was shown as mean ± SEM. from three parallel experiments. **c**, CCK8 proliferation assay of LL2 and H460 cells treated by escalating doses of SB225002 (0-10 μM) for 24 h, 48 h, and 72 h (left) and corresponding IC50 concentration of SB225002 (right). Data was shown as mean ± SD. **d**, Flow cytometric analyses of PI-Annexin V staining of apoptotic cells treated by different concentration SB225002 (0, 0.5, 1, and 5 μM) for 24 h. Cells for Annexin V+/PI− and Annexin V+/PI+ were both considered apoptotic. Data was shown as mean ± SEM, *n* = 3. **e**, Immunofluorescent staining of TUNEL of LL2 and H460 cells treated by different concentration of SB225002 (0, 500 nM, or 1 μM) for 24 h (left), and quantification of TUNEL-positive cells (right). Scale bar, 25 μm. TUNEL-stained cells are in green, DAPI-stained nuclei are in blue. Using 0.1% DMSO as control. Data was shown as mean ± SEM, *n* = 3. IC50, half maximal inhibitory concentration, *p < 0.05, **p < 0.01, ***p < 0.001, ns represents *p*>0.05

The result of flow cytometric analysis demonstrated that CXCR2 was obviously expressed in murine cell line, LL2 (Lewis lung carcinoma, LLC) cell and human lung cancer cell line, A549, PC-9, and H460 (Fig. 2a). Meanwhile, CXCR2-associated chemokines, such as CXCL1, CXCL2, CXCL3, CXCL5, CXCL6, CXCL7, CXCL8 and MIF, were also expressed by those cells (Fig. 2b). Based on the expression levels of them, murine cell line LL2 and human cell line H460 were chosen for further analyses. Selective inhibitor of CXCR2, SB225002, was confirmed to inhibit the proliferation of lung cancer cells in a both time-dependent and dose-dependent manner. The 50% inhibitive concentrations (IC50) of SB225002 on LL2 cells and H460 cells for 24 h were 785.6 nM and 1263 nM, respectively (Fig. 2c). SB225002 was also capable to induce lung cancer cells apoptosis in a dose-dependent manner via flow cytometric analysis (Fig. 2d). TUNEL staining further confirmed that SB225002 promoted lung cancer cells apoptosis (Fig. 2e). All these data suggested that CXCR2 could promote lung cancer cells proliferation and anti-apoptosis.

### Blockade of CXCLs/CXCR2 promotes lung cancer cell senescence and inhibits CXCR2-associated EMT through p38/ERK MAPK pathway

**Fig. 3:**
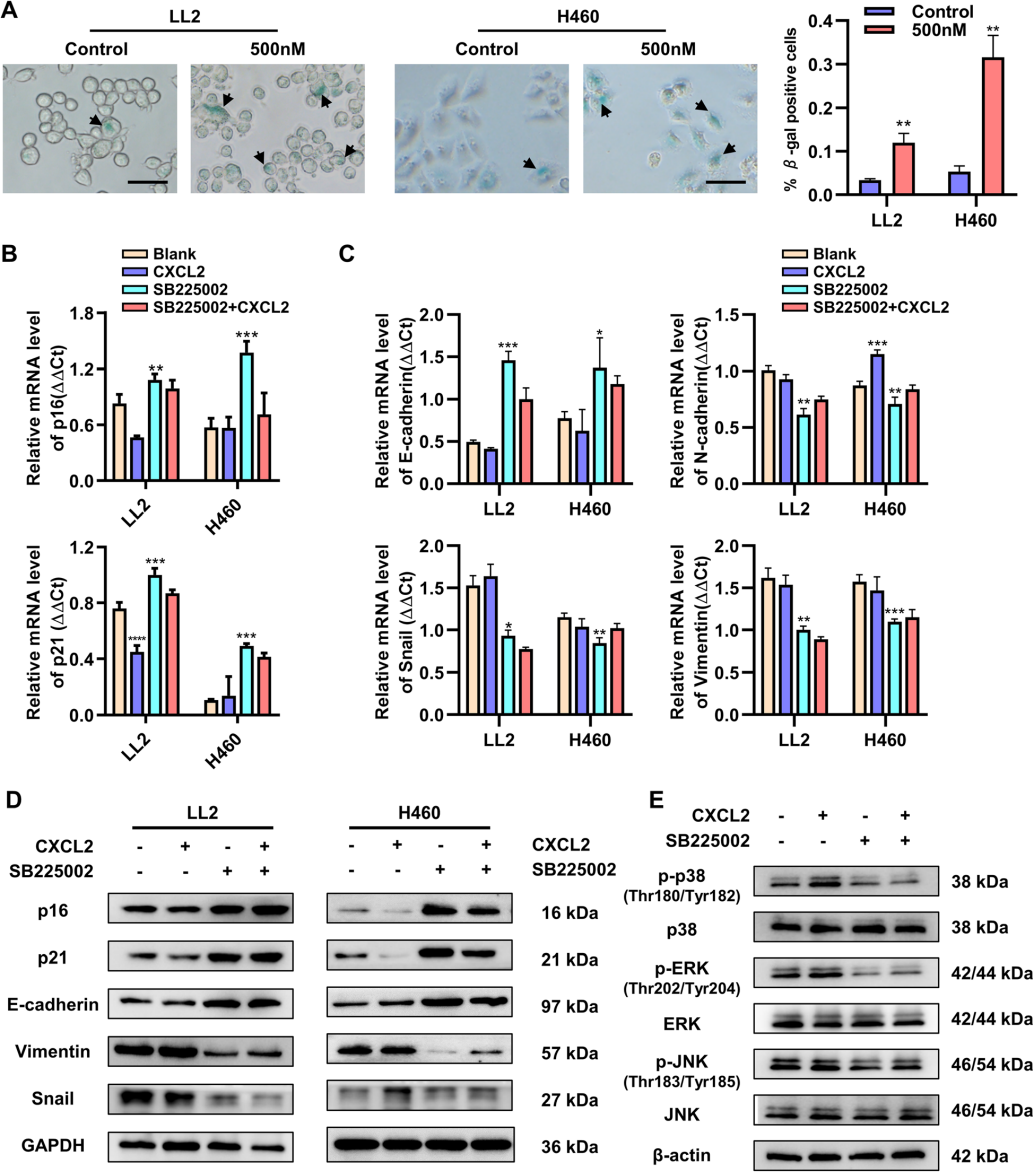
Blockade of CXCLs/CXCR2 axis promotes lung cancer cell senescence and inhibits CXCR2-associated EMT through MAPK pathway. **a**, β-galactosidase staining of LL2 and H460 cells treated by SB225002 (0 and 500 nM) for 24 h (left) and quantification of β-galactosidase staining positive cells (right). Arrows point β-galactosidase staining positive cells. Scale bar, 50 μm, data was shown as mean ± SEM, *n* = 3. **b**-**d**, LL2 and H460 cells were treated by CXCL2 (100 ng/ml) or SB225002 (500 nM) for 24 h, and the total mRNA and proteins were collected for qRT-PCR and Western blot analyses. Senescence-related markers p16 and p21 (**b**) and EMT-associated markers (E-cadherin, N-cadherin, Snail and Vimentin) (**c**) were verified via relative mRNA expression level. Detection of protein level was confirmed by Western blot (**d**). Data was shown as mean ± SEM. from three parallel experiments. **e**, Western blot analyses of p38/ERK/JNK MAPK pathway in LL2 cells. β-actin and GAPDH were used as a loading control. Data was shown as mean ± SEM. from three parallel experiments. *p < 0.05, **p < 0.01, ***p < 0.001, ns represents *p*>0.05

Tumor cells usually show the ability of unlimited proliferation and avoiding aging. When cells go through senescence, expression of senescence-associated β-galactosidase (SA-β-gal) is up-regulated. To investigate the role of CXCR2 in tumor cell senescence, the expression of SA-β-gal was detected while using SB225002 on LL2 and H460 cells. After 24 h, the result showed that the percentage of β-gal-positive cells in SB225002 group was higher than that of control group (Fig. 3a). The senescence-associated markers p16 and p21 were up-regulated after using SB225002 and down-regulated after using CXCL2 (Fig. 3b). Epithelial to mesenchymal transition (EMT) is thought to be closely associated with tumor metastasis, and CXCR2 is reported to regulate cellular EMT. QRT-PCR was carried out to detect the expression of EMT-associated markers, such as E-cadherin, N-cadherin, Snail, and Vimentin, which were differentially altered after administration of SB225002 or CXCL2 (Fig. 3c). Western blotting was performed to confirm the results of qRT-PCR (Fig. 3d). MAPK pathway usually is important for cellular activities. After using SB225002 and CXCL2 on LL2 cells, p38/ERK MAPK signaling pathway was found involved in CXCR2-associated signaling pathway. The levels of p38 and ERK proteins phosphorylation were obviously altered, whereas p-JNK almost remained unchanged during activation or inhibition of CXCR2 (Fig. 3e).

In our experiments, by using CXCR2 inhibitor SBB225002, CXCLs/CXCR2 axis was found to promoted lung cancer cells proliferation and EMT, and inhibited lung cancer cells apoptosis and senescence via p38/ERK, not JNK, MAPK pathway.

### CXCR2^+^ TANs significantly infiltrated into tumor tissue of LL2-bearing mice

**Fig. 4:**
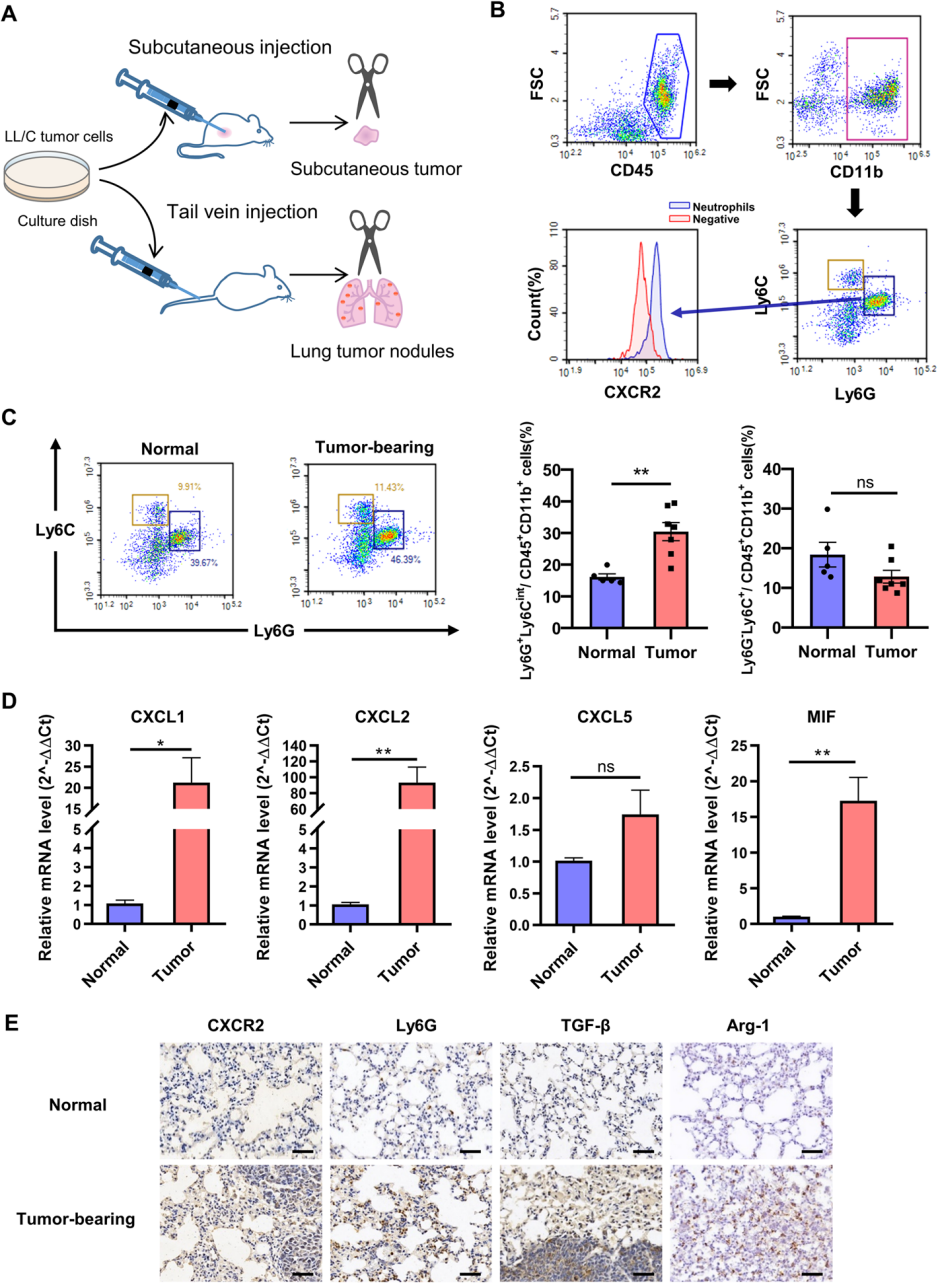
CD11b^+^Ly6C^mid^Ly6G^hi^CXCR2^+^ neutrophils significantly infiltrate into tumor tissue of LL2-bearing mice. **a**, Construction of lung orthotopic model and subcutaneous tumor model in mice, *n* = 6–7. For lung orthotopic model, 5 × 10^5 LL2 cells were injected into tail vein of C57BL/6 mice. For subcutaneous tumor model, 2 × 10^6 LL2 cells were injected into the right flank of C57BL/6 mice. At the endpoint of experiments, lung tumor nodules and tumor masses were collected for further analyses. **b**, Flow cytometric characterization of neutrophils (CD45^+^CD11b^+^Ly6C^mid^Ly6G^high^CXCR2^+^) and monocytes (CD45^+^CD11b^+^Ly6C^high^Ly6G^−^) from tumor tissues. CXCR2-unstained cells were used for negative control. **c**, Flow cytometric analyses of neutrophils and monocytes infiltrated in the tumor microenvironment of normal and tumor-bearing mice (left) and quantification of infiltrated immune cells (right). Data was shown as mean ± SEM, *n* = 6–7. **d**, The relative mRNA expression of CXCL1, CXCL2, CXCL5, and MIF in the lung of normal and tumor-bearing mice. Data was shown as mean ± SEM, *n* = 6–7. **e**, Immunohistochemistry staining of neutrophils’ markers CXCR2 and Ly6G, and immune suppressive markers TGF-β and Arg-1 in the lung of normal and tumor-bearing mice. Scale bar, 50 μm. *p < 0.05, **p < 0.01, ***p < 0.001, ns represents *p*>0.05

In previous experiment, the expression of CXCR2 both on tumor cells and in tumor stroma was noticed by IHC analysis. CXCR2 is a chemokine receptor mainly expressed on neutrophils surface. To investigate the infiltration of CXCR2^+^ neutrophils in mouse model of lung cancer, orthotopic lung cancer model and subcutaneous tumor model were established via tail vein injection or subcutaneous injection of LL2 cells (Fig. 4a). Neutrophils (CD45^+^CD11b^+^Ly6C^mid^Ly6G^high^) and monocytes (CD45^+^CD11b^+^ Ly6C^high^ Ly6G^−^) were two groups of abundant myeloid cells in tumor microenvironment. Meanwhile, those neutrophils expressed chemokine receptor CXCR2 (Fig. 4b). The result of flow cytometric analyses of the tumor microenvironment of mouse lung cancer mode showed that the percentage of CXCR2-positive neutrophils were significantly increased, whereas the percentage of monocytes remained unchanged (Fig. 4c). Meanwhile, the expressions of CXCR2-associated chemokines (CXCL1, CXCL2, CXCL5 and MIF) were up-regulated in lung tumor tissues, which indicated the involvement of CXCR2 chemotaxis in neutrophil infiltration (Fig. 4d). Tumor microenvironment is usually an immune-suppressive environment and promotes tumor growth. By IHC analysis, the increased expression of CXCR2 and neutrophil infiltration were both confirmed in lung cancer. Immune-suppressive factors, TGF-β and Arg-1, were also up-regulated in lung cancer at the same time (Fig. 4e). TGF-β is known to induce polarization of neutrophils to N2 type. N2 TANs usually secreted more TGF-β and Arg-1, which lead to an immunosuppressive environment and contribute tumor cell immune escape [12].

### Selective targeting of CXCR2 reduces tumor growth in orthotopic lung cancer model and subcutaneous tumor model

**Fig. 5:**
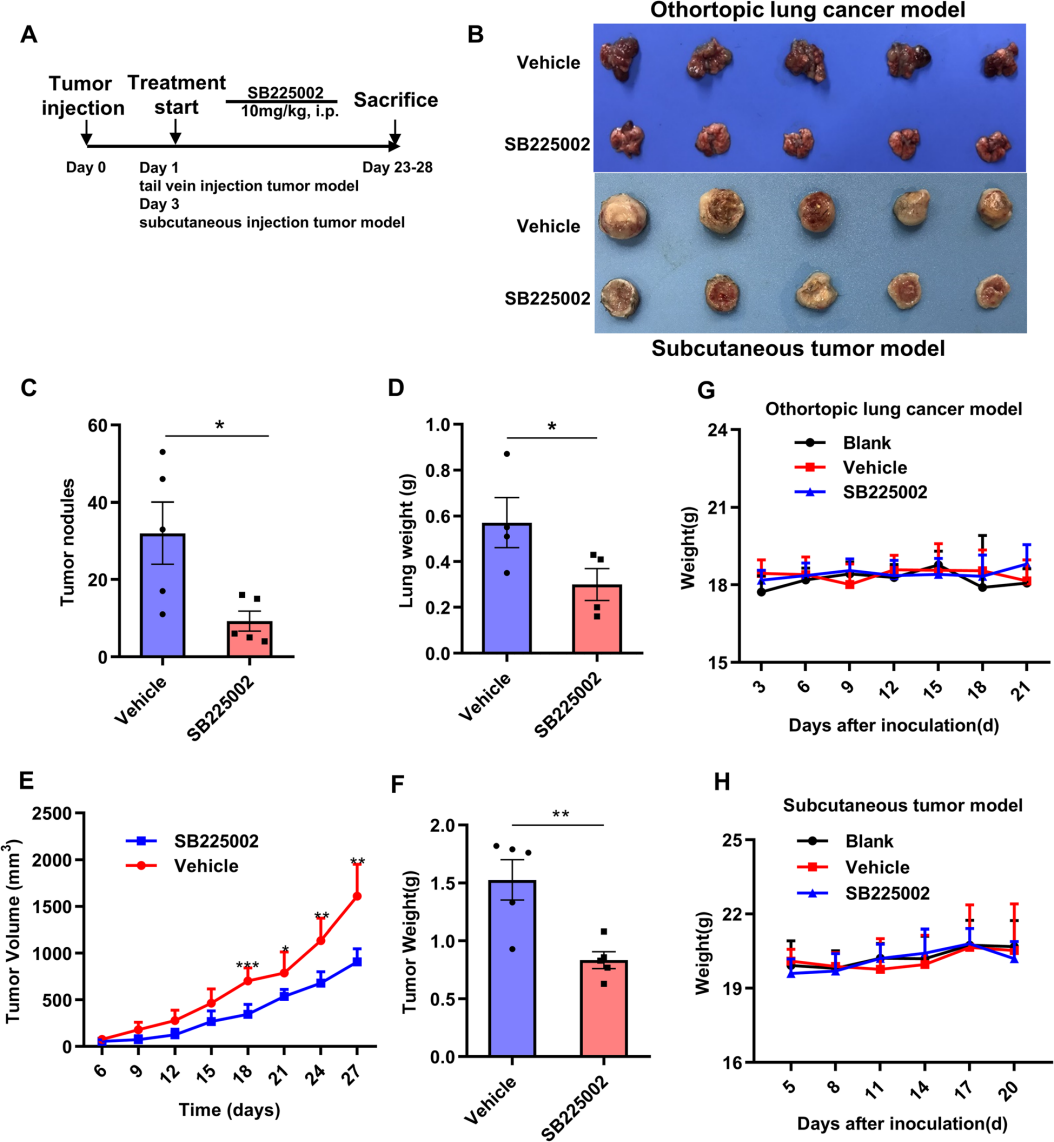
Selective targeting of CXCR2 reduces tumor growth in LL2 orthotopic lung cancer and subcutaneous tumor models. **a**, Therapy regimen of the experiment. CXCR2 inhibitor SB225002 was administered at 10 mg/kg by intraperitoneal (i.p.) injection every day, *n* = 6–7. **b**, Tumors from vehicle- and SB225002-treated groups of lung orthotopic model (upper panel) and subcutaneous tumor model (down panel) at the end point of experiment, respectively. **c**-**d**, Tumor nodules in the lung and lung weight of the orthotopic model treated with vehicle or SB225002, Data was shown as mean ± SD, *n* = 5. **e**-**f**, Tumor growth curves and tumor weight at the sacrifice of subcutaneous xenograft model. Data was shown as mean ± SD, *n* = 5. **g**-**h**, Body weight changes of the mice from orthotopic model (**g**) and subcutaneous xenograft model (**h**). Data was shown as mean ± SD, *n* = 5. *p < 0.05, **p < 0.01, ***p < 0.001, ns represents *p*>0.05

Based on the features of the tumor microenvironment in lung cancer models, we reasoned that pharmacological CXCR2 inhibition might lead to anti-tumor response via regulation of neutrophils infiltration and repression of tumor cells. To confirm the therapeutic effect of CXCR2 inhibition, SB225002, a selective CXCR2 inhibitor, was used in bothorthotopic lung cancer model and subcutaneous tumor model (Fig. 5a). SB225002 treatment alone led to an obvious reduction in tumor growth of both orthotopic lung cancer model and subcutaneous tumor model (Fig. 5b). At the end point of orthortopic model, tumor nodules in the lung of SB225002 group were fewer than those of vehicle group (Fig. 5c). Total lung weights of SB225002 group were also lighter than those of vehicle group (Fig. 5d). In subcutaneous tumor model, obvious tumor growth inhibition was observed in SB225002 group (Fig. 5e). After sacrifice of mice, the subcutaneous tumor weights from each group were shown in Fig. 5f. Furthermore, to test the potential side effects of SB225002, the body weights were measured and serum biochemical analysis and vital organ HE staining were performed for each experimental group. The result showed no obvious difference in mouse weight between SB225002 (10 mg/kg, every day) group and control groups (Fig. 5g and h). The serum biochemical analysis demonstrated that, except for a slight increase (no statistical difference) in ASL and ALT, biochemical parameters of SB225002 group did not altered compared to the control groups (Supplementary Fig. 2A). Meanwhile, HE staining of vital organ showed no obvious organ damage in SB225002 group (Supplementary Fig. 2B). Therapeutic evaluation and tolerability study of SB225002 indicated that SB225002 (10 mg/kg, every day) showed promising anti-tumor effects with high safety.

### Inhibition of CXCR2 decreases neutrophils with suppressive phenotype infiltration in tumor microenvironment

**Fig. 6:**
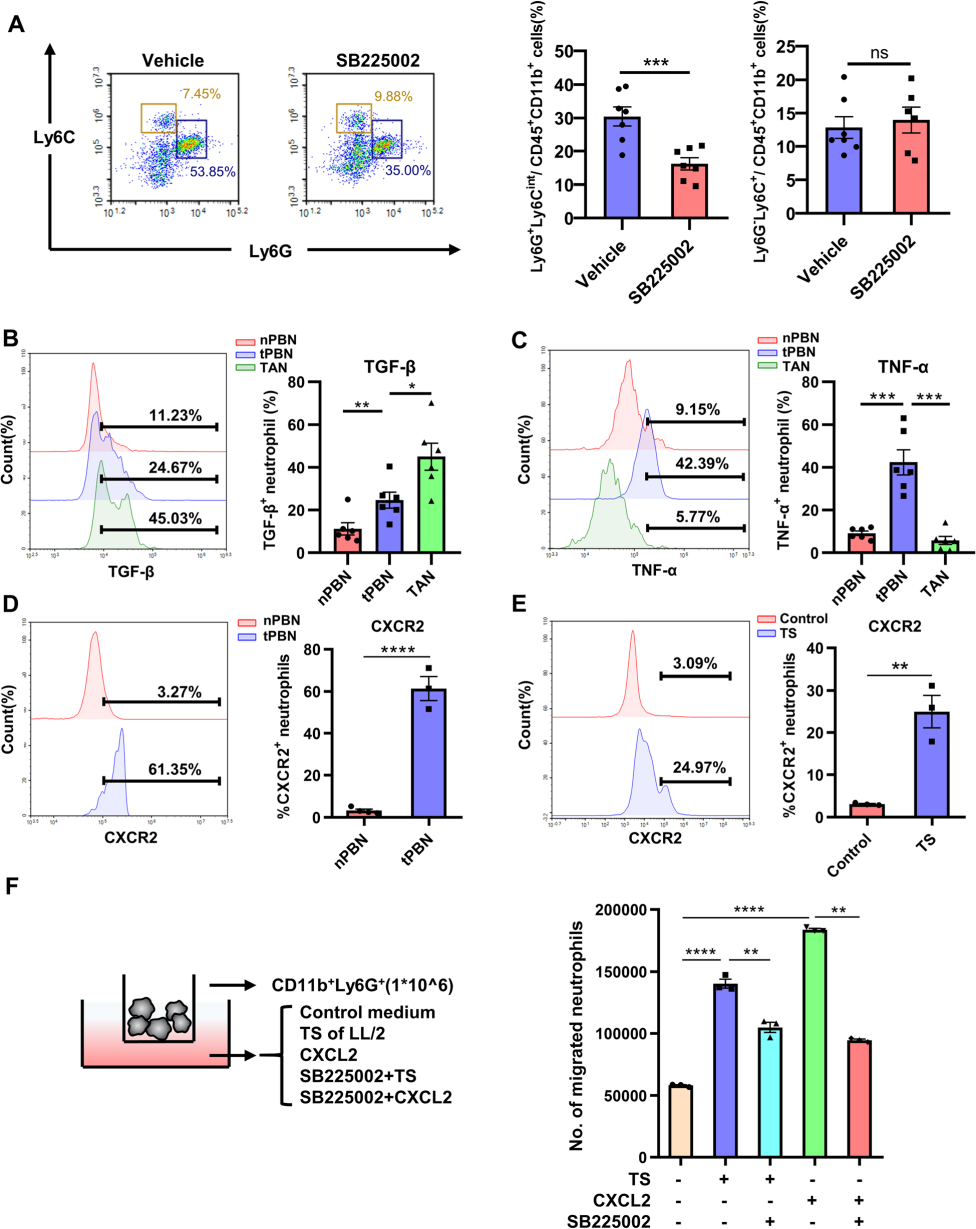
Inhibition of CXCR2 decreases infiltration of suppressive neutrophils in the tumor microenvironment. **a**, Lung tissues of mice from orthotopic lung cancer model treated with vehicle or SB225002 (10 mg/kg) were collected and digested for flow cytometric analyses of infiltrated neutrophils (CD45^+^CD11b^+^Ly6C^mid^Ly6G^high^) and monocytes (CD45^+^CD11b^+^Ly6C^+^Ly6G^−^) in tumor microenvironment. Data was shown as mean ± SD, *n* = 6–7. **b**-**c**, Neutrophils (CD45^+^CD11b^+^Ly6C^mid^Ly6G^high^) from tumor microenvironment (TAN), peripheral blood of tumor-bearing mice (tPBN), and peripheral blood of normal mice (nPBN) were further analyzed. TGF-β (**b**) and TNF-α (**c**) produced by neutrophils of three groups were determined by flow cytometry. Data was shown as mean ± SD, *n* = 6–7. **d**, Expression of CXCR2 on the surface of nPBN and tPBN. Data was shown as mean ± SEM from three parallel experiments, *n* = 3. **e**, Primary neutrophils were extracted from the bone marrow of healthy mice, and then seeded in plates and treated by tumor supernatant (TS) or RPIM 1640 medium. Expression of CXCR2 on the surface of neutrophils was detected by flow cytometry. Data was shown as mean ± SEM from three parallel experiments, *n* = 3. **f**, 1 × 10^6 primary neutrophils were seeded into the upper chamber (3 μM), and RPIM 1640 medium, LL2 cell tumor supernatant, chemokine CXCL2 (50 ng/ml), or SB225002 (500 nM) were added into the bottom chamber (left)). After 6 h of incubation, the number of migrated neutrophils was counted by flow cytometry (right). Data was shown as mean ± SEM from three parallel experiments, *n* = 3. *p < 0.05, **p < 0.01, ***p < 0.001, ns represents *p*>0.05

To further investigate the mechanisms involved in CXCR2 inhibitor-induced anti-tumor effects, we analyzed the neutrophils infiltration into tumor microenvironment. Flow cytometry analysis discovered that neutrophils (CD11b^+^Ly6C^mid^Ly6G^high^) were significantly decreased after treated with SB225002 while monocytes (CD11b^+^Ly6C^high^ Ly6G^−^) remained unchanged (Fig. 6a). Blockade of CXCLs/CXCR2 using selective CXCR2 inhibitor SB225002 could effectively decrease the infiltration of neutrophils in tumor microenvironment. Tumor associated neutrophils (TANs) are usually divided into two different types, N1 and N2 type. Neutrophils of N1 type are anti-tumor type and secrete many pro-inflammatory factors such as TNF-α and produce ROS and H_2_O_2_ to kill tumor cells [13, 39, 40]. Type N2 TANs are known to have significantly decreased abilities in killing tumors and they secrete anti-inflammatory factors such as TGF-β and Arg-1 [14]. The expression level of TGF-β in TANs was much higher than that in neutrophils from peripheral blood of normal mice (nPBN) or from peripheral blood of tumor-bearing mice (tPBN) (Fig. 6b). Meanwhile TANs secreted less TNF-α compared with nPBN and tPBN (Fig. 6c). The chemotaxis of neutrophils from peripheral blood to tumor microenvironment is based on CXCLs/CXCR2. We further found expression level of CXCR2 on tPBN surface was much higher than that on nPBN surface (Fig. 6d), which might lead to enhanced chemotaxis of neutrophils in tumor. The result of IHC suggested inhibition of CXCR2 could decrease neutrophil infiltration and attenuate immunosuppression in tumor microenvironment with decreased levels of TGF-β and Arg-1 (Supplementary Fig. 3). In vitro, LL2 cells were cultured for 24 h, and the primary tumor supernatant was harvest and centrifuged to remove dead cells. Then we isolated neutrophils from mice bone marrow and treated with centrifuged tumor supernatant (TS) with a ratio of 1:1. The result of flow cytometry analysis demonstrated that TS could effectively up-regulate the expression level of CXCR2 on neutrophil surface (Fig. 6e). The chemotaxis of CXCLs/CXCR2 axis was enhanced by tumor stimulation, and we established a co-culture system with Transwell membranes (3 μm). Isolated neutrophils were seeded in upper chamber, and 1640 RPMI medium, TS, CXCL2 or SB225002 were added to the lower chamber (Fig. 6f left). After 6 h of incubation, the number of migrated neutrophils was significantly elevated by adding TS and CXCL2, and SB225002 effectively inhibited TS and CXCL2- induced neutrophils migration (Fig. 6f right). Collectively, these results revealed that recruitment of neutrophils with immune-suppressive phenotype to tumor microenvironment was mediated by CXCLs/CXCR2 axis and SB225002.

### Blockade of CXCR2 enhances anti-tumor T cell activity via promoting CD8^+^ T cell activation

**Fig. 7:**
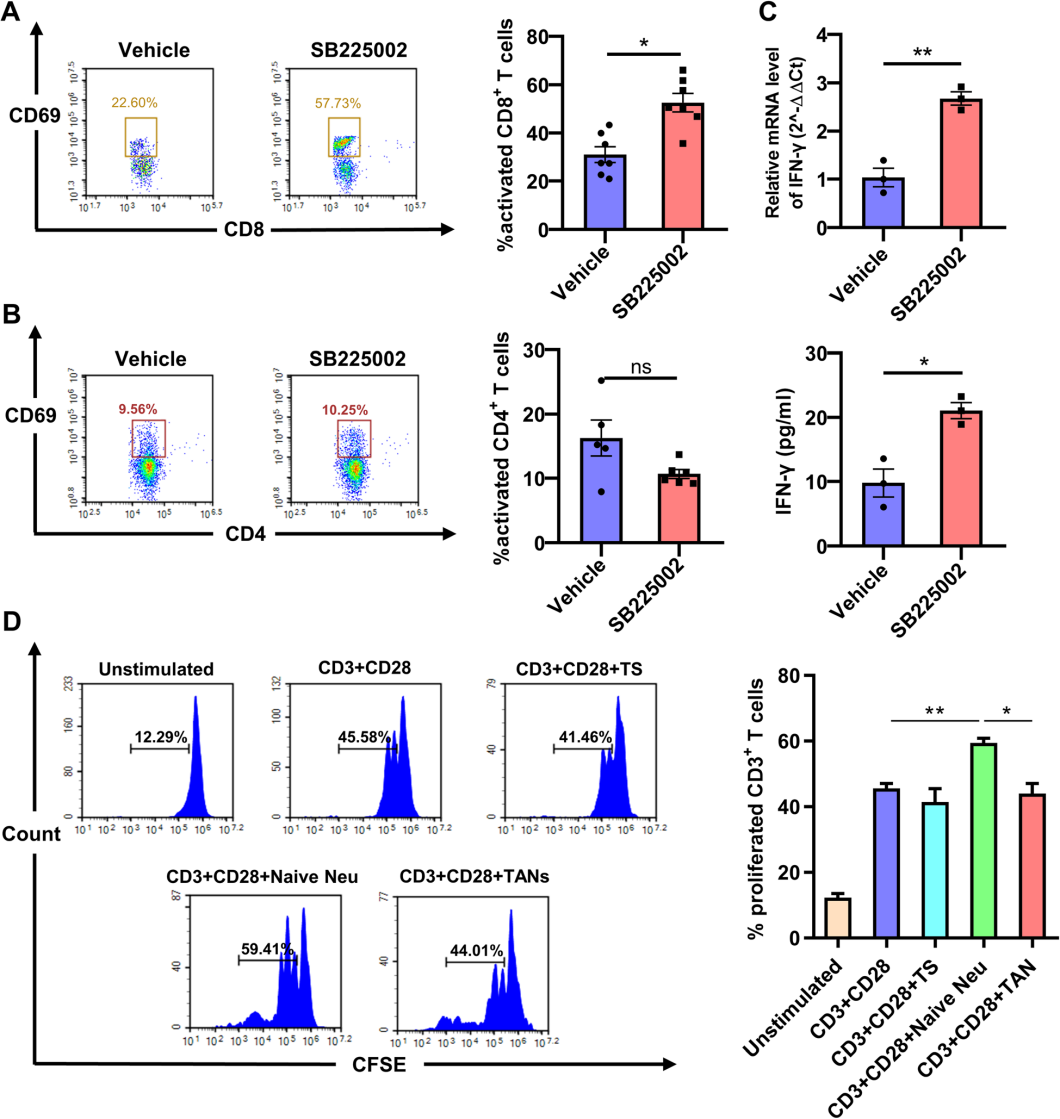
Blockade of CXCR2 enhances anti-tumor T cell activity via promoting CD8^+^ T cell activation. Lung tissues of orthotopic model mice treated with vehicle or SB225002 (10 mg/kg) were collected and digested. **a**-**b**, Flow cytometric analyses (left) and quantification (right) of activated CD8^+^ T cells (CD8^+^CD69^+^) and CD4^+^ T cells (CD4^+^CD69^+^) from the tumor microenvironment of SB225002- or vehicle-treated LL2 tumor-bearing mice. Data was shown as mean ± SD, *n* = 6–7. **c**, Expression of IFN-γ in tumor microenvironment was detected by qRT-PCR (upper panel) and ELISA (down panel). Data was shown as mean ± SEM from three parallel experiments, *n* = 3. **d**, Primary T lymphocytes were extracted from the spleen of healthy mice, and then were stained with CFSE and co-cultured with tumor supernatant (TS), primary neutrophils (naïve neutrophils), or TANs (left). Anti-CD3 and anti-CD28 antibodies were added to stimulate T cells proliferation. Quantification of T cells proliferation rate (right). Data was shown as mean ± SEM from three parallel experiments, *n* = 3. *p < 0.05, **p < 0.01, ***p < 0.001, ns represents *p* > 0.05. Neu, neutrophil; TAN, tumor-associated neutrophil; CFSE, carboxy fluorescein diacetate succinimidyl ester

Neutrophils are known to interact with T cells via antigen presentation and cytokine secretion. For example, Arg-1 is mainly secreted by tumor-infiltrating myeloid cells, including TANs, and can directly suppress T lymphocytes and participate in tumor immune escape [41, 42]. In our current study, we found that after administration of SB225002, activated CD8^+^ T lymphocytes were obviously elevated (Fig. 7a). Whereas activated CD4^+^ T lymphocytes remained unchanged (Fig. 7b). Furthermore, we detected the level of IFN-γ secreted in tumor tissues. The expression level of IFN-γ was obviously up-regulated in SB225002 treatment group (Fig. 7c). To investigate the relationship between neutrophils and T lymphocytes, T cells were extracted from mouse spleen and labeled with CFSE. These T cells were stimulated by anti-CD3 and anti-CD28 antibody and then co-cultured with TS, naïve neutrophils and TS-treated neutrophils (TANs). Neutrophils isolated from mouse bone marrow were able to stimulate T cell proliferation. However, those neutrophils, after co-cultured with TS, lost the ability to stimulate T cells proliferation (Fig. 7d). These data indicated that, neutrophils infiltrated into tumor microenvironment were polarized to a suppressive phenotype. TANs then suppressed anti-tumor immune responses via affecting the function of T lymphocytes, mainly CD8^+^ T lymphocytes.

### SB225002 and cisplatin (DDP) show combined treatment efficacy on lung cancer

**Fig. 8:**
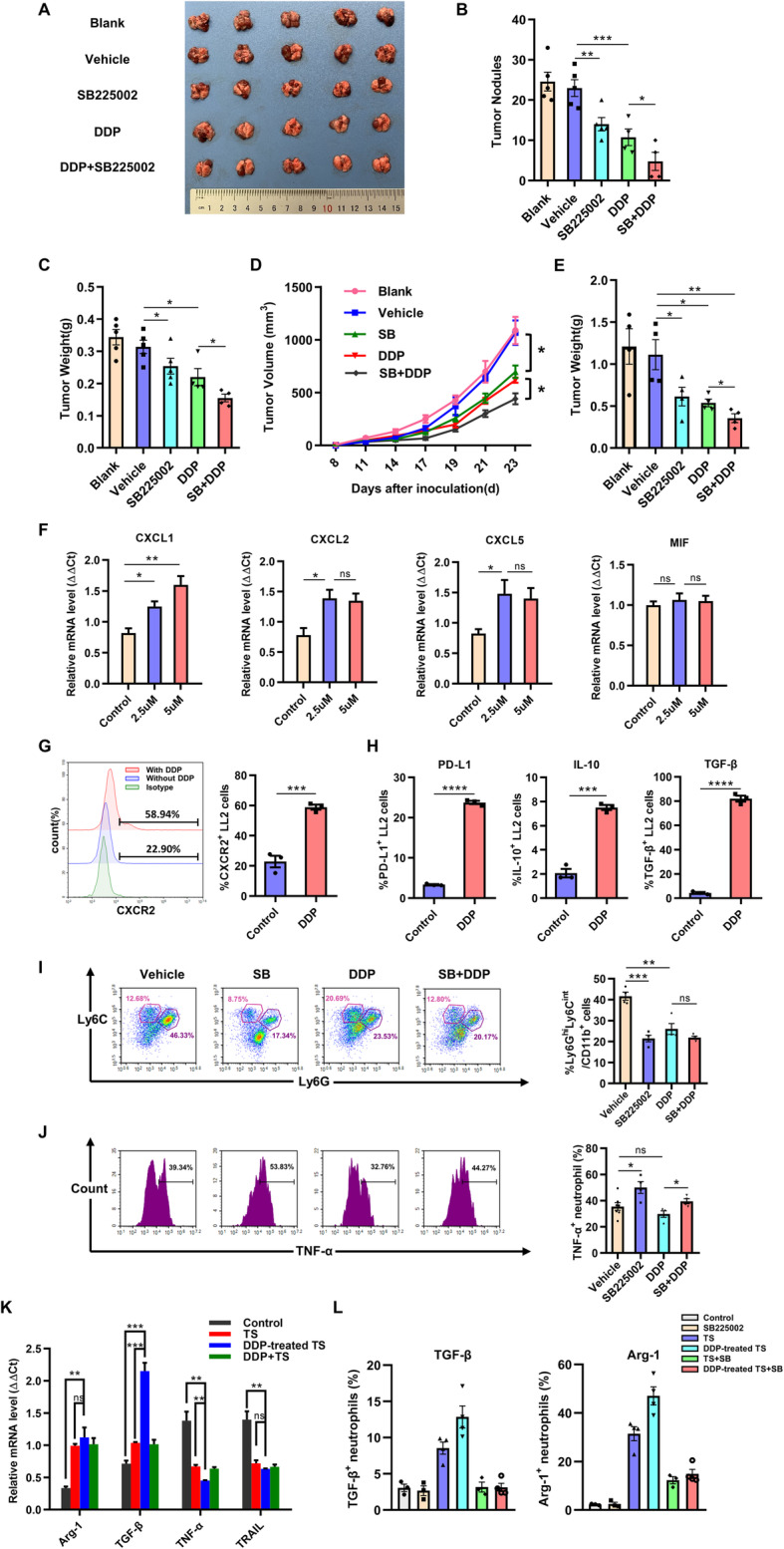
SB225002 and cisplatin shows combined treatment efficacy on lung cancer. LL2-bearing mice were randomly divided into 5 groups: blank control, solvent control group (vehicle, 25% PEG 400 + 5% Tween 80 + 69%ddH2O + 1%DMSO), SB225002 group (10 mg/kg once a day), cisplatin group (DDP, 2.5 mg/kg once a week), and SB225022 combined with DDP group. Each group contained 6–7 mice. **a**-**c**, For lung orthotopic tumor model, the representative picture of lung was shown (**a**), and the number of pulmonary nodules (**b**) and total lung weight (**c**) were counted. Data was shown as mean ± SD, *n* = 5. **d**-**e**, For subcutaneous tumor model, the tumor growth curves (**d**) and mean tumor weights at the end point of this experiment (**e**) were recorded. Data was shown as mean ± SD, *n* = 4–5. **f**, LL2 cells were treated by cisplatin (2.5 μM and 5 μM) for 24 h and collected. The relative mRNA expression of CXCL1, CXCL2, CXCL3 and MIF of LL2 cells following cisplatin treatment. Data was shown as mean ± SEM from three parallel experiments, *n* = 3. **g**, The expression of CXCR2 on LL2 cells surface following cisplatin treatment was detected by flow cytometry. Data was shown as mean ± SEM from three parallel experiments, *n* = 3. **h**, The levels of immunosuppressive molecules of LL2 cells following cisplatin treatment, including PD-L1, IL-10, and TGF-β, were measured by flow cytometry. Data was shown as mean ± SEM from three parallel experiments, *n* = 3. **i**, Flow cytometric analyses of neutrophils infiltrated into tumor microenvironment of mice from 5 experimental groups (left) and quantification of infiltrated neutrophils (right). Data was shown as mean ± SD, *n* = 4–5. **j**, The expression level of TNF-α secreted by neutrophils by flow cytometric analyses (left) and quantification of TNF-α level (right). Data was shown as mean ± SD, *n* = 4–5. **k**, The relative mRNA expressions of Arg-1, TGF-β, TNF-α and TRAIL of neutrophils after TS or DDP-treated TS treatment. Data was shown as mean ± SEM from three parallel experiments, *n* = 3. **l**, Based on the treatment of Fig. 8k, SB225002 of 500 nM was further administrated. The expressions of Arg-1, TGF-β of neutrophils were detected by flow cytometry. Data was shown as mean ± SEM from three parallel experiments, *n* = 3. SB, SB225002; TS, tumor supernatant. *p < 0.05, **p < 0.01, ***p < 0.001, ns represents *p* > 0.05

It is reported that the expression of CXCR2 is increase after the administration of platinum-based drugs [35, 43]. We further used combined therapy of SB225002 and cisplatin to treat LL2 cells orthotopic lung cancer model and subcutaneous tumor model. In orthotopic lung cancer model, compared with 23.0 lung nodules in mice of vehicle-treated group, administration of SB225002 or cisplatin either reduced tumor nodules in the lung (14 and 10.75, respectively), and mice of combined-therapy group showed fewer nodules (4.75, Fig. 8a and b). Combined therapy also showed inhibition effects on tumor growth by in orthotopic lung cancer model (Fig. 8c). In subcutaneous tumor model, combined therapy of SB225002 and cisplatin inhibited the tumor growth compared with other 4 groups (Fig. 8d). Tumor growth curves indicated that combined-therapy group mice showed an obvious reduction in tumor growth compared with other groups (Fig. 8e). SB225002 combined with cisplatin showed synergistic anti-tumor effect in lung cancer.

After observing the combined therapeutic effect of SB225002 and cisplatin, we hypothesized that CXCLs/CXCR2 signaling was changed following cisplatin treatment. In vitro, LL2 cells were co-cultured with cisplatin for 24 h. The expression levels of CXCR2-associated chemokines, such as CXCL1, CXCL2, and CXCL5, not MIF, in LL2 cells were significantly increased following cisplatin treatment (Fig. 8f). The expression of CXCR2 on LL2 cells surface was significantly up-regulated following cisplatin stimulation (Fig. 8g). Combination of SB225002 and DDP obviously synergistically inhibited proliferation and anti-apoptosis of LL2 cell line (Supplementary Fig. 4). Meanwhile, tumor cells are known to secrete immunosuppressive cytokines or express immunosuppressive markers to modulate tumor microenvironment and escape from anti-tumor immune response. The expression levels of PD-L1, IL-10 and TGF-β in LL2 cells were significantly elevated after treated with cisplatin (Fig. 8h). Though CXCR2 on tumor cells was up-regulated followed DDP treated, blockade of CXCR2 couldn’t alter the expression of those immunosuppressive markers (Supplementary Fig. 5). The result of flow cytometry analysis indicated that cisplatin could also decrease the infiltration of neutrophils compared with control group (Fig. 8i). However, in the tumor microenvironment of cisplatin-treated group, the remaining neutrophils expressed the same level of TNF-α as vehicle-treated group. After added SB225002, TNF-α secreted by neutrophils was significantly up-regulated compared with cisplatin-alone group (Fig. 8j). In vitro, we collected tumor supernatant of cisplatin-treated LL2 cells (DDP-treated TS) and then co-cultured with neutrophils. The expression levels of Arg-1 and TGF-β in neutrophils were up-regulated after TS and DDP-treated TS stimulation. Furthermore, neutrophils following DDP-treated TS stimulation secreted more Arg-1 and TGF-β and less TNF-α and TRAIL compared with TS-treated group (Fig. 8k). We further administrated SB225002 to confirm the impact of CXCR2 on TANs. Flow cytometry analysis showed inhibition of CXCR2 could suppress the expression of Arg-1 and TGF-β of DDP-treated TS-treated TANs, which indicated that CXCR2 receptor might affect the activation of neutrophils (Fig. 8l). These results suggested that CXCLs/CXCR2 signaling was up-regulated after cisplatin stimulation and cisplatin enhanced the immune-suppression of tumor microenvironment.

## Discussion

The 5-year survival of non-small cell lung cancer patients is less than 20%, and it is obviously required to improve anti-tumor therapy for lung cancer [44]. Platinum-based chemotherapy is the primary treatment for lung cancer. However, drug-tolerance and poor response of patients limit the effective use of platinum-based drugs [5]. The results of this study indicate that CXCR2 is overexpressed in the tumor tissues of patients with lung adenocarcinoma and squamous cell lung cancer and high expression of CXCR2 is associated with poor prognosis. CXCLs/CXCR2 autocrine loop exist in lung cancer cells and participate in the regulation of apoptosis, proliferation, senescence, and EMT of tumor cells through p38/ERK MAPK pathway. In mice of lung cancer model, blockade of CXCR2 inhibits lung tumor growth via decreasing immune suppressive neutrophils infiltration, augmenting the activation of CD8^+^ T cells and improved therapeutic effect of cisplatin by modulating tumor microenvironment.

Mounting evidence supports the important role of CXCR2 in tumorigenesis and cancer patients’ prognosis. Up-regulation of CXCR2 has been found in various cancers, such as ovarian carcinoma, pancreatic carcinoma and breast cancer [45–47]. In the present study, CXCR2 was overexpressed in lung cancer tissues and associated with poor overall survival of patients with NSCLC. Lung cancer cells continuously secrete CXCR2-associated chemokines, and high level expressions of those chemokines promote tumor progression and metastasis [29, 30, 48]. We employed mouse Lewis lung carcinoma cell line and human lung cancer cell line H460, which expressed CXCR2 on the surface and secreted CXCR2-associated chemokines, such as CXCL1, CXCL2, CXCL5 and MIF. Our results support that CXCLs/CXCR2 chemokine autocrine loop contributes to tumor cellular activities, such as proliferation, apoptosis, senescence and EMT [46, 49, 50]. It’s worth noting that the role of CXCR2 played in cellular senescence is complicated. Some studies have suggested expression of CXCR2 maintained the oncogenic senescence signal and promoted cellular senescence [51, 52]. Our results indicated cellular senescence could be inhibited by CXCLs/CXCR2 signal [46]. However, due to oncogenic role of CXCR2 in a lot of cancers, we infer that CXCLs/CXCR2 signal is essential for normal/preneoplastic cells to maintain response to oncogenic senescence signal. During the progression of cancer, oncogene-induced senescence lose the inhibition of carcinogenesis. Blockade of CXCLs/CXCR2 signal might indirectly induce senescence of tumor cell. Activation of CXCR2 leads to the phosphorylation of ERK and p38 but not of JNK, and MAPK signaling is usually associated with cell proliferation and survival [28]. The data makes blockade of CXCR2 a promising anti-tumor strategy [31, 53].

Another important role of CXCR2 is to regulate neutrophils migration to tumor microenvironment, and neutrophils lacking CXCR2 are preferentially retained in bone marrow [54]. Neutrophils of N1 type produce more NET (neutrophils extracellular traps), ROS (reactive oxygen species) and secrete TNF-α to kill tumor cells and produce H_2_O_2_ to destroy the environment for tumor growth, inhibiting tumor metastasis [13, 39, 40]. Whereas, the expression levels of these secreted factors are relatively low in N2 neutrophils. N2 neutrophils show decreased ability to kill tumor cells and produce TGF-β and Arg-1 to inhibit anti-tumor immune response and promote tumor growth [14]. Study has found neutrophils infiltrated into tumor microenvironment gradually lost the features of N1 neutrophil and promoted tumor progression [55]. In lung cancer model of this study, CXCR2-associated chemokines were significantly up-regulated and the expression of CXCR2 on surface of neutrophils was increased following tumor stimulation. Pharmacological inhibition of CXCR2 significantly decreased lung cancer progression with ameliorated recruitment of neutrophils into tumor microenvironment.

Neutrophils are known to interact with T cells via antigen presentation and cytokine secretion [41, 42]. Our results demonstrated the activated CD8^+^ T cells were increased after SB225002 treatment in lung cancer model. Primary neutrophils are capable to promote T cells proliferation, which is consist with our results [41, 56]. However, neutrophils lost the capacity to stimulate T lymphocytes proliferation after co-cultured with tumor supernatant. These data suggested CXCR2 targeted therapy could inhibit lung cancer progression via decreasing the infiltration of suppressive neutrophils and augmenting the activation of T cells.

Platinum-based anti-tumor drugs are primary chemotherapeutic agents for lung cancer. CXCLs/CXCR2 axis has been reported to affect therapeutic effects of platinum-based drugs. In the treatment of prostate cancer with oxaliplatin, the expression of CXCL1, CXCL8 and CXCR2 were significantly increased, while CXCR2 inhibitors could enhance the cytotoxicity of oxaliplatin and achieve the combined anti-tumor effect [35]. The inhibitors of CXCLs/CXCR2 axis could make recurrent, refractory tumors sensitive to chemotherapy again [32–34]. In our experiment, SB225002 combined with cisplatin showed combined therapeutic effect in lung cancer. The expression of CXCR2 and CXCR2-associated chemokines of tumor cells were significantly increased following cisplatin stimulation. Meanwhile, cisplatin could reduce neutrophils infiltration into tumor microenvironment, which was in accordance with former study [57, 58]. However, those remaining neutrophils secreted less TNF-α compared with SB225002-treated group, acting as N2 type. Therefore, we hypothesized that cisplatin could promote immune suppression of tumor through increasing the expression of suppressive markers in tumor cells. Clinical trials have also studied the immunogenic effect of platinum-based therapy via combination of platinum with ICB therapy and showed promising therapeutic effects [59]. In vitro experiments, the expressions of PD-L1, IL-10 and TGF-β in tumor cells were increased after stimulation of cisplatin. TGF-β is known to polarize neutrophils to N2 type and has a strong immunosuppressive effect [12, 60–62].

The results of this study highlighted the changes of tumor microenvironment following chemotherapy or administration of CXCR2 inhibitor. The development of ICB therapy for cancer treatment is a landmark, including for NSCLC. Up-regulation of PD-L1 on tumor cells or MDSCs contributes a lot to tumor immune escape and meanwhile affects the therapeutic effect of ICB therapy [63]. Progression of cancer is usually accompanied with infiltration of MDSCs which makes a delayed treatment of anti-PD1 less benefit [64]. Preclinical studies have showed promising results of combined therapy of CXCR2 antagonists and PD-1 inhibitor [28, 65, 66]. Therefore, selective inhibition of CXCR2 shows promising prospect in improvement of current anti-tumor therapy.

Currently, several CXCR2 antagonists have been investigated in clinical trials [67]. Due to the potent inhibition for neutrophils recruitment, CXCR2 inhibitors were initially used in respiratory diseases and gradually in cancer. Six CXCR2 antagonists have been investigated in clinical trials, including AZD5069, Reparixin, Danirixin, SB-656933, Navarixin, and SX-682. Despite abundant preclinical experiments results, there is no CXCR2 antagonists approved for use in clinic.

## Conclusions

**Fig. 9:**
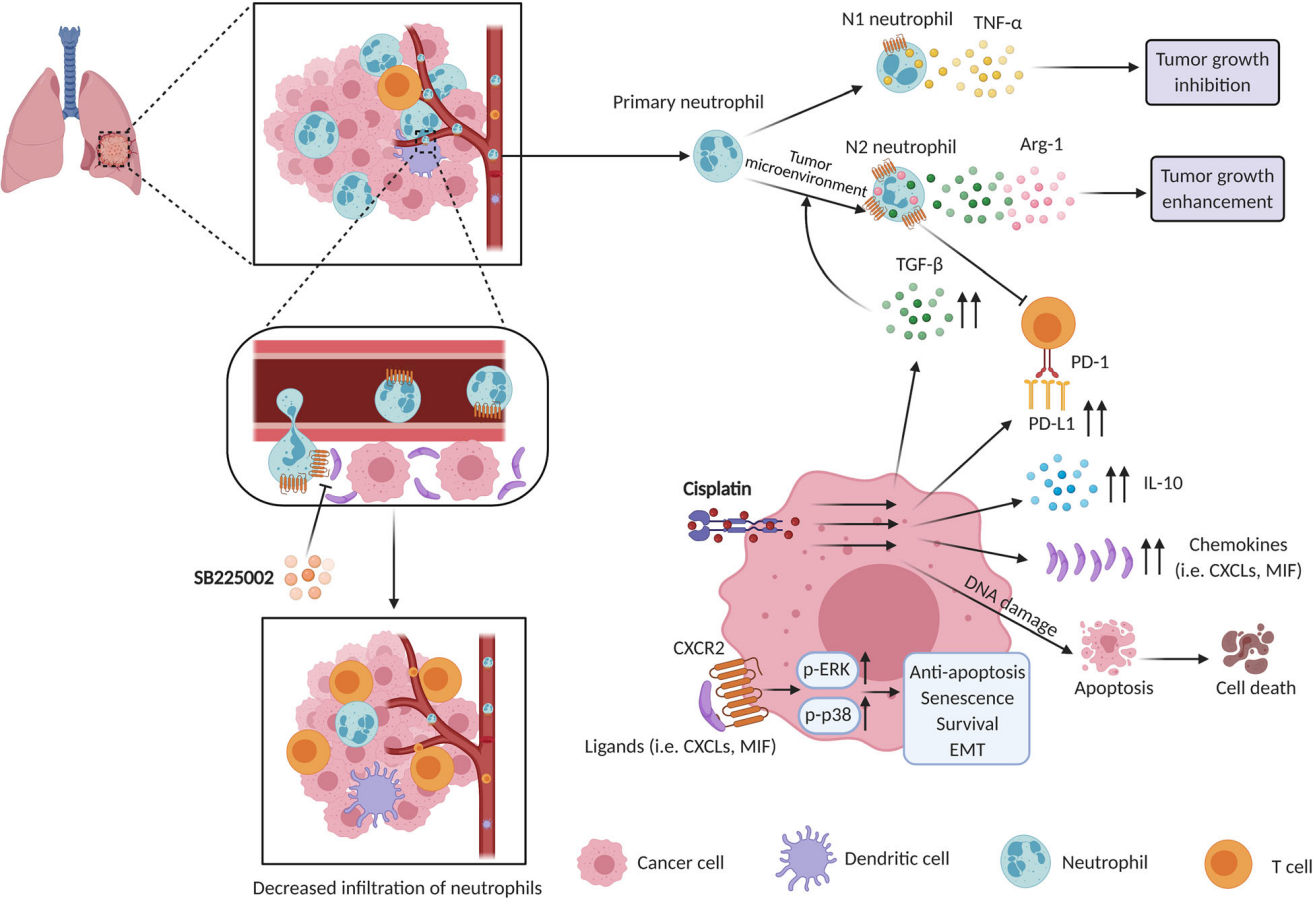
Schematic diagram of the mechanistic findings. CXCR2 showed potent chemotaxis on neutrophils. Tumor microenvironment was abundant in immunosuppressive cytokines, such as TGF-β and Arg-1, which induced polarization of neutrophils to pro-tumor N2 type and impaired anti-tumor immune response. SB225002, a selective inhibitor of CXCR2, could effectively inhibit neutrophils infiltration to tumor microenvironment. Cisplatin, a common chemotherapy agent for lung cancer, could induce up-regulation of immunosuppressive markers, such as TGF-β, IL-10, and PD-L1 in tumor cells, which might contribute to reduced anti-tumor effect

Several studies have explored the role of CXCLs/CXCR2 axis in lung cancer. Nevertheless, it is unclear what role CXCR2 and its chemokines are playing in the therapy of lung cancer. In this research, we found CXCR2 expression level was significantly associated with prognosis and survival of patients with lung cancer. Targeting CXCR2 obviously inhibited tumor growth, with decreased infiltration of TANs and increased response of T cells. Our findings confirm the anti-cancer effects and safety of CXCR2 targeted therapy. Meanwhile, based on our study, blockade of CXCR2 could improve the traditional therapy against lung cancer, such as cisplatin (Fig. 9).

## Supplementary Information

**Additional file 1: Supplementary Fig. 1.** Expression of CXCR2 in lung cancer patients. A, Representative immunohistochemical staining for CXCR2 in lung cancer patients. Scale bar, 25um. B, Positive rate of CXCR2 in lung cancer patients.

**Additional file 2: Supplementary Fig. 2.** Safety and tolerance of SB225002 in the treatment of lung cancer. A, Serum biochemical detection of liver function, kidney function, myocardial function and other important markers of tumor-bearing mice treated by vehicle or SB225002. Data was shown as mean ± SD, *n* = 5. B, Histological examinations of vital organs from tumor-bearing mice by HE staining. Images are presented at a magnification of 200× for liver, 100× for spleen, kidney, and heart. *p < 0.05, **p < 0.01, ***p < 0.001, ns represents *p* > 0.05

**Additional file 3: Supplementary Fig. 3.** Infiltration of neutrophils and the levels of immune-related molecules in vehicle- versus SB225002- treated group. A, After treatment of SB225002, neutrophils infiltration and the expression of TGF-β in tumor microenvironment of both lung orthotopic cancer model and subcutaneous tumor model were detected by IHC. Scale bar, 20 μm. B, The relative mRNA expression levels of Arg-1, TGF-β, and TNF-α in the tumor microenvironment of lung orthotopic cancer model. Data was shown as mean ± SEM from three parallel experiments, *n* = 3. *p < 0.05.

**Additional file 4: Supplementary Fig. 4.** Combination of SB225002 and DDP inhibits LL2 cell line proliferation and promotes it apoptosis. LL2 cells were treated by cisplatin (2.5 μM) or SB225002 (500 nM) for 24 h. A, Flow cytometric analyses of apoptotic LL2 cells stained with PI-Annexin V. B, Quantification analyses of apoptotic cells (Annexin V-positive cells). C, Proliferation curves of LL2 cells tested by CCK8 assay. Data was shown as mean ± SEM from three parallel experiments, *n* = 3. D. Combination index (CI) values at combined doses determined by CompuSyn. CI values less than 1.0 indicated synergism. *p < 0.05, **p < 0.01, ***p < 0.001, ns represents *p*>0.05.

**Additional file 5: Supplementary Fig. 5.** The impact of SB225002 on LL2 cells treated by cisplatin. A-C, The expression of PD-L1 (A), IL-10 (B), and TGF-β (C) detected by flow cytometry. *p < 0.05, **p < 0.01, ***p < 0.001, ns represents *p*>0.05.

**Additional file 6: Supplementary Table 1.** Primers for quantitative real-time PCR.

## Data Availability

The datasets in this study are available from the corresponding author on reasonable request.

## Abbreviations

EMT: Epithelial-to-mesenchymal transition
TANs: Tumor-associated neutrophils
NSCLC: Non-small cell lung cancer
ROC: Receiver operating characteristic
CCK-8: Cell counting kit-8
ELISA: Enzyme-linked immunosorbent assay
PI: Propidium iodide
TUNEL: TdT-mediated dUTP Nick-End Labeling
qRT-PCR: Quantitative real-time PCR
TS: Tumor supernatant
SA-β-gal: Senescence-associated β-galactosidase
nPBN: Neutrophils from peripheral blood of normal mice
tPBN: Neutrophils from peripheral blood of tumor-bearing mice
DDP: Cisplatin
SB: SB225002
IRS: Immunoreactivity scoring
