# Supplementary figures and images for: Targeting CXCR2 inhibits the progression of lung cancer and promotes therapeutic effect of cisplatin
Source: Mol Cancer. 2021 Apr 4;20:62. doi: 10.1186/s12943-021-01355-1 (PMC8019513; doi:10.1186/s12943-021-01355-1)

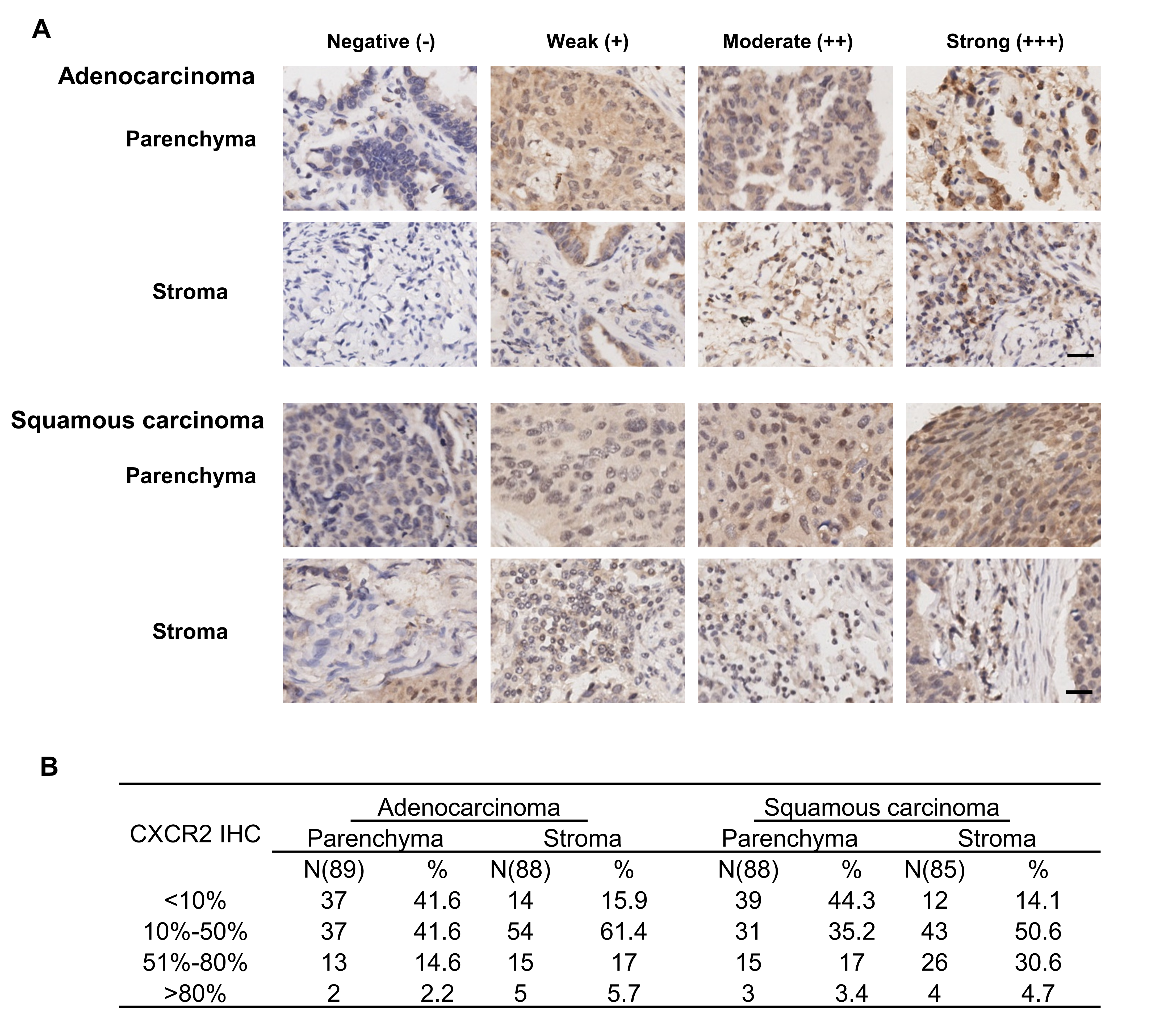

Supplement: Supplementary file 1 — Additional file 1: Supplementary Fig. 1. Expression of CXCR2 in lung cancer patients. A, Representative immunohistochemical staining for CXCR2 in lung cancer patients. Scale bar, 25um. B, Positive rate of CXCR2 in lung cancer patients. [file 12943_2021_1355_MOESM1_ESM.tif]

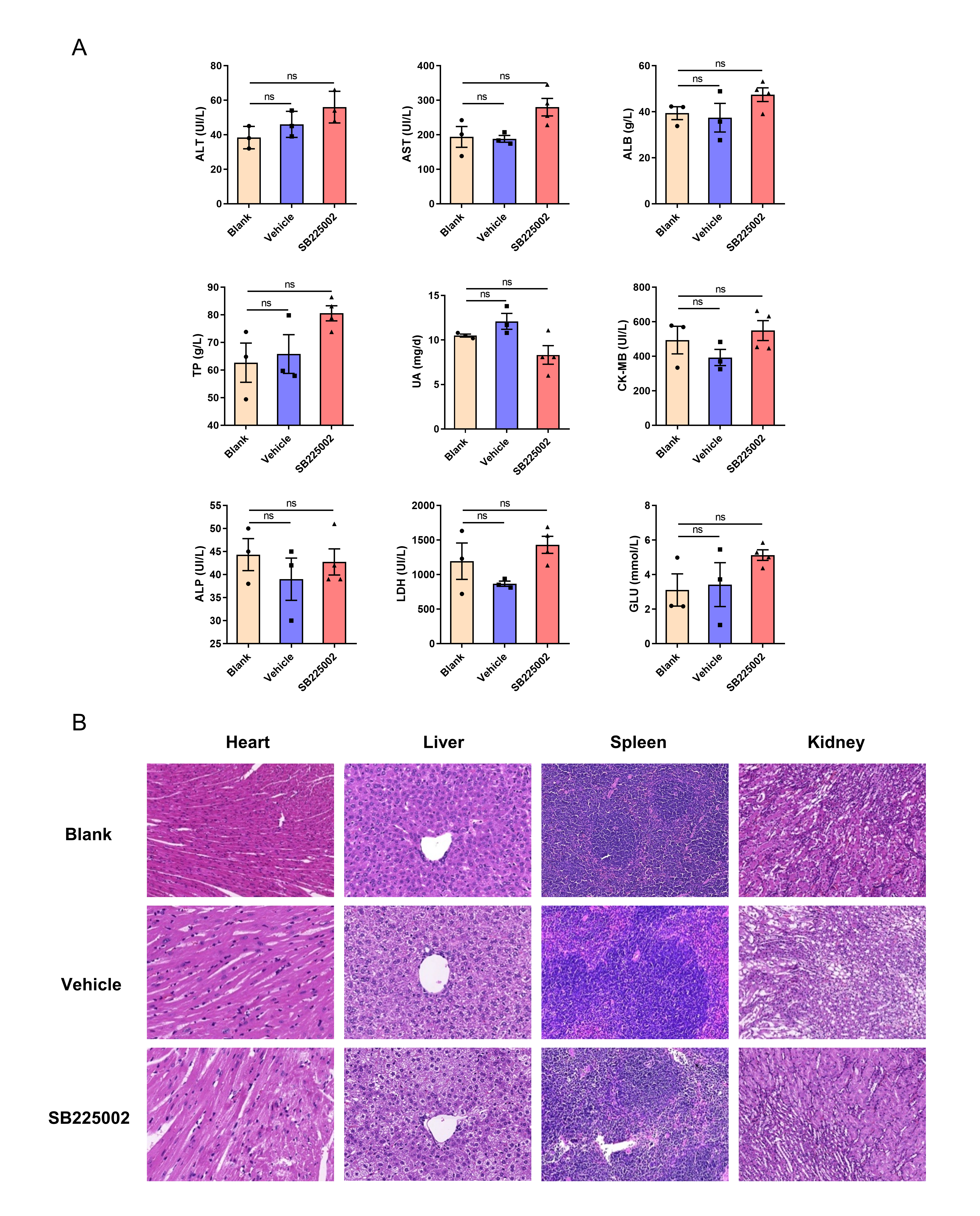

Supplement: Supplementary file 2 — Additional file 2: Supplementary Fig. 2. Safety and tolerance of SB225002 in the treatment of lung cancer. A, Serum biochemical detection of liver function, kidney function, myocardial function and other important markers of tumor-bearing mice treated by vehicle or SB225002. Data was shown as mean ± SD, n = 5. B, Histological examinations of vital organs from tumor-bearing mice by HE staining. Images are presented at a magnification of 200× for liver, 100× for spleen, kidney, and heart. *p < 0.05, **p < 0.01, ***p < 0.001, ns represents p > 0.05 [file 12943_2021_1355_MOESM2_ESM.tif]

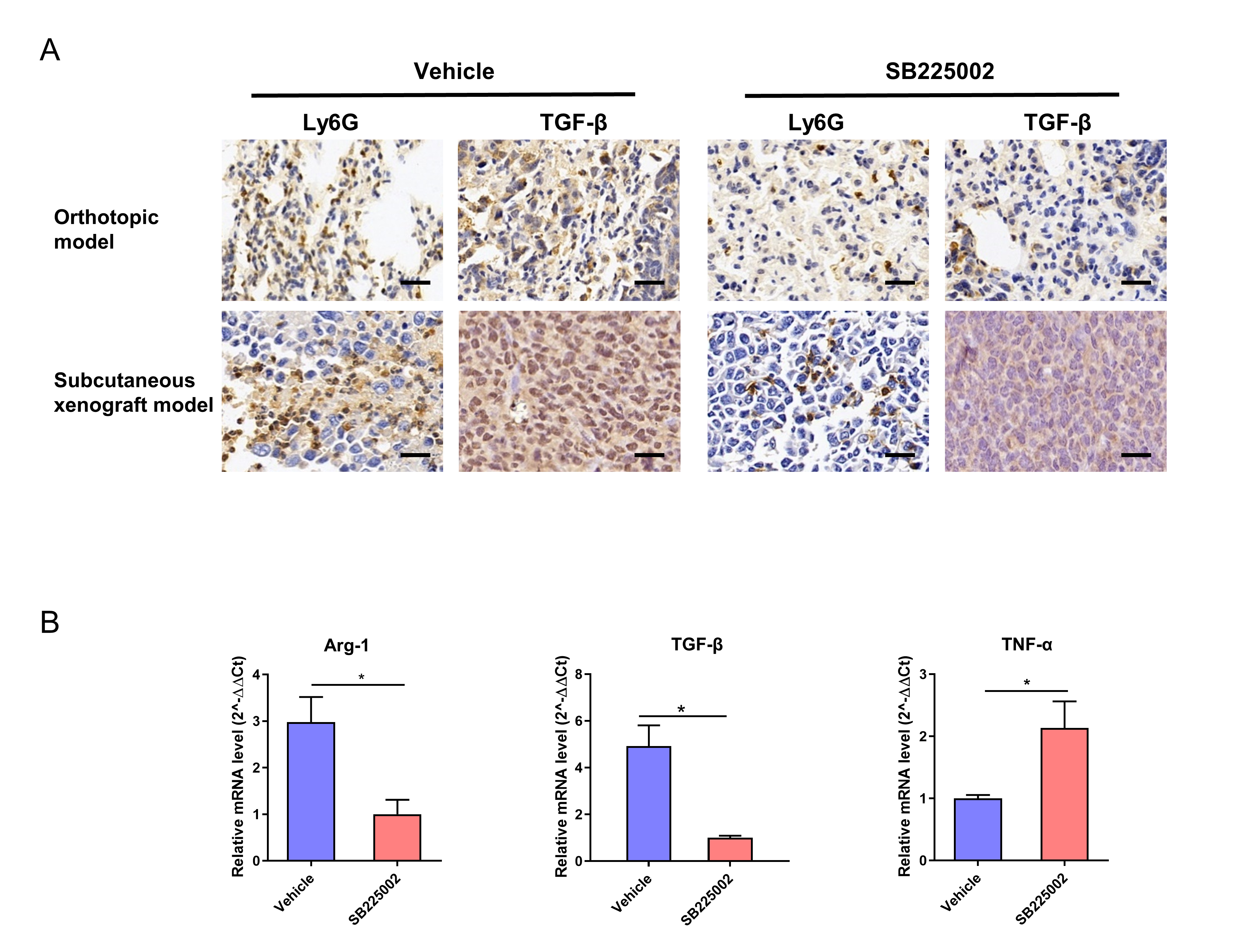

Supplement: Supplementary file 3 — Additional file 3: Supplementary Fig. 3. Infiltration of neutrophils and the levels of immune-related molecules in vehicle- versus SB225002- treated group. A, After treatment of SB225002, neutrophils infiltration and the expression of TGF-β in tumor microenvironment of both lung orthotopic cancer model and subcutaneous tumor model were detected by IHC. Scale bar, 20 μm. B, The relative mRNA expression levels of Arg-1, TGF-β, and TNF-α in the tumor microenvironment of lung orthotopic cancer model. Data was shown as mean ± SEM from three parallel experiments, n = 3. *p < 0.05. [file 12943_2021_1355_MOESM3_ESM.tif]

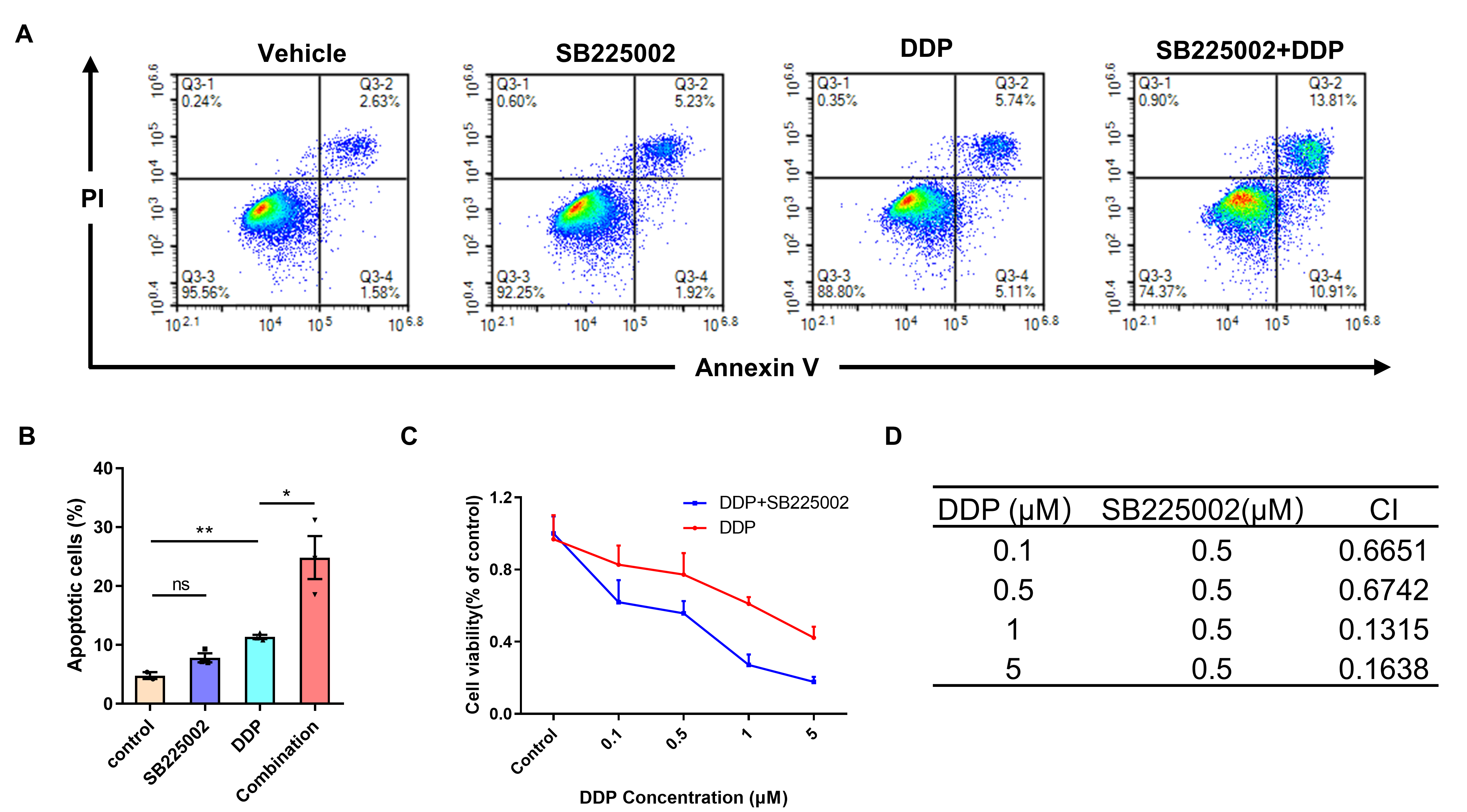

Supplement: Supplementary file 4 — Additional file 4: Supplementary Fig. 4. Combination of SB225002 and DDP inhibits LL2 cell line proliferation and promotes it apoptosis. LL2 cells were treated by cisplatin (2.5 μM) or SB225002 (500 nM) for 24 h. A, Flow cytometric analyses of apoptotic LL2 cells stained with PI-Annexin V. B, Quantification analyses of apoptotic cells (Annexin V-positive cells). C, Proliferation curves of LL2 cells tested by CCK8 assay. Data was shown as mean ± SEM from three parallel experiments, n = 3. D. Combination index (CI) values at combined doses determined by CompuSyn. CI values less than 1.0 indicated synergism. *p < 0.05, **p < 0.01, ***p < 0.001, ns represents p>0.05. [file 12943_2021_1355_MOESM4_ESM.tif]

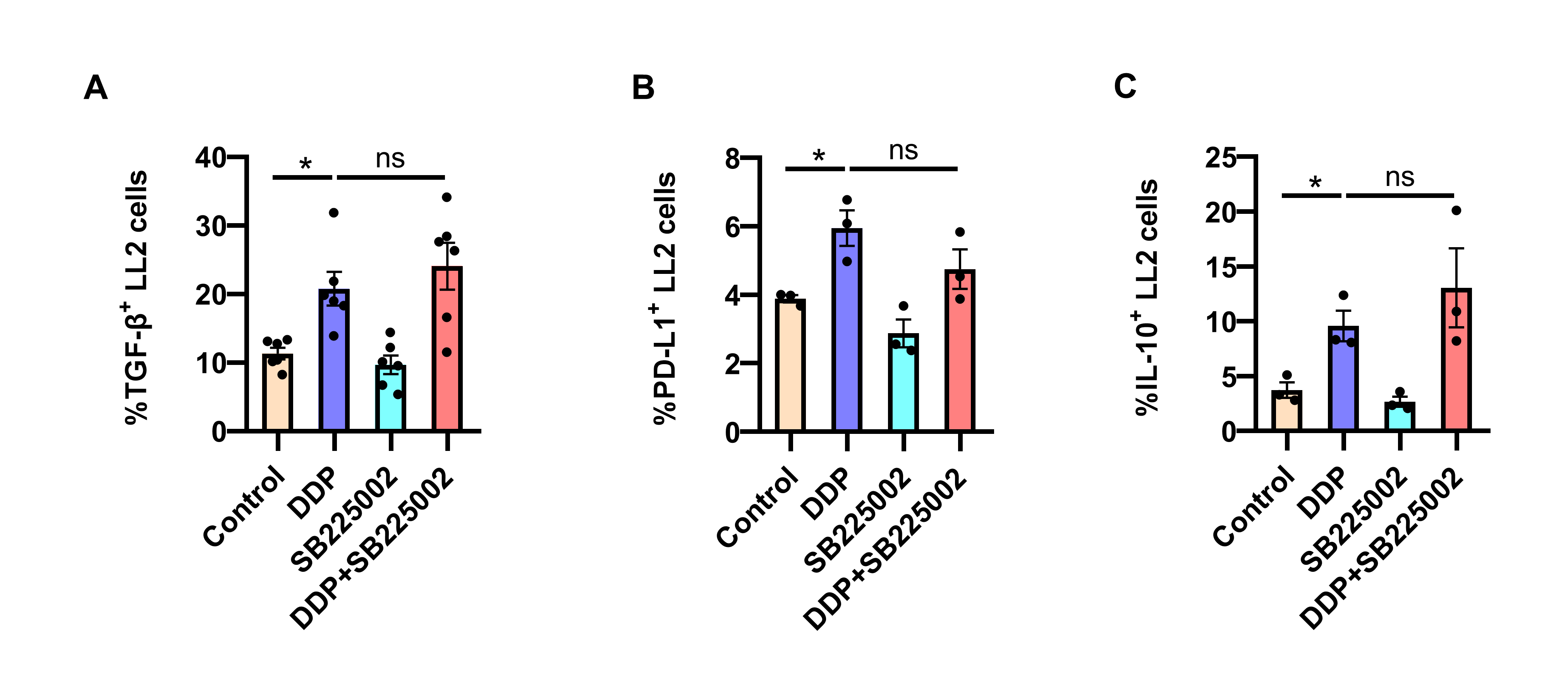

Supplement: Supplementary file 5 — Additional file 5: Supplementary Fig. 5. The impact of SB225002 on LL2 cells treated by cisplatin. A-C, The expression of PD-L1 (A), IL-10 (B), and TGF-β (C) detected by flow cytometry. *p < 0.05, **p < 0.01, ***p < 0.001, ns represents p>0.05. [file 12943_2021_1355_MOESM5_ESM.tif]
