# Supplementary material for: Targeting CXCR2 inhibits the progression of lung cancer and promotes therapeutic effect of cisplatin
Source: Mol Cancer. 2021 Apr 4;20:62. doi: 10.1186/s12943-021-01355-1 (PMC8019513; doi:10.1186/s12943-021-01355-1)
Supplement: Supplementary file 6 — Additional file 6: Supplementary Table 1. Primers for quantitative real-time PCR. [file 12943_2021_1355_MOESM6_ESM.docx]

**Supplementary Table 1.** Primers for quantitative real-time PCR.

| Species | Gene name |  | Primers |
| --- | --- | --- | --- |
| Mouse | CXCL1 | forward | 5’- CTG GGA TTC ACC TCA AGA ACA TC -3’ |
|  |  | reverse | 5’- CAG GGT CAA GGC AAG CCT C-3’ |
|  | CXCL2 | forward | 5’- GAA GTC ATA GCC ACT CTC AAG G -3’ |
|  |  | reverse | 5’- CTT CCG TTG AGG GAC AGC-3’ |
|  | CXCL3 | forward | 5’- GAAGTCATAGCCACTCTCAAGG -3’ |
|  |  | reverse | 5’- AAAGACACATCCAGACACCG -3’ |
|  | CXCL5 | forward | 5’- GTT CCA TCT CGC CAT TCA TGC -3’ |
|  |  | reverse | 5’- GCG GCT ATG ACT GAG GAA GG-3’ |
|  | CXCL6 | forward | 5’- GCGTTTCTGTTGCTGTTCAC -3’ |
|  |  | reverse | 5’- AGTTTAGCTATGACTTCCACCG -3’ |
|  | CXCL7 | forward | 5’- TTCCCATTGAGCATTGTTAT -3’ |
|  |  | reverse | 5’- TGCTTGACTCCAGGCGATTT -3’ |
|  | MIF | forward | 5’-CCA GAA CCG CAA CTA CAG TAA-3’ |
|  |  | reverse | 5’-CCG GTG GAT AAA CAC AGA AC-3’ |
|  | CXCR1 | forward | 5’- CAATGGCCGAGGCTGAATA -3’ |
|  |  | reverse | 5’- GAAGGGACACCAGTGCATAAAA -3’ |
|  | CXCR2 | forward | 5’- TCT TCC AGT TCA ACC AGC C-3’ |
|  |  | reverse | 5’- ATC CAC CTT GAA TTC TCC CAT C-3’ |
|  | E-cadherin | forward | 5’- AAG TGA CCG ATG ATG ATG CC -3’ |
|  |  | reverse | 5’- CTT CAT TCA CGT CTA CCA CGT -3’ |
|  | N-cadherin | forward | 5’- TGAAACGGCGGGATAAAGAG -3’ |
|  |  | reverse | 5’- GGCTCCACAGTATCTGGTTG -3’ |
|  | Snail | forward | 5’- CACACGCTGCCTTGTGTCT -3’ |
|  |  | reverse | 5’- GGTCAGCAAAAGCACGGTT -3’ |
|  | Vimentin | forward | 5’- ACG ATC TCA CCC TCA GGG CT -3’ |
|  |  | reverse | 5’- GGG TCG CTG AGT CAG TGG AT -3’ |
|  | p16 | forward | 5’- GAACTCTTTCGGTCGTACCC -3’ |
|  |  | reverse | 5’- CGAATCTGCACCGTAGTTGA -3’ |
|  | p21 | forward | 5’- TTGTCGCTGTCTTGCACTCT -3’ |
|  |  | reverse | 5’- TCTCTTGCAGAAGACCAATC -3’ |
|  | Arginase-1 | forward | 5’- CAG AAG AAT GGA AGA GTC AG -3’ |
|  |  | reverse | 5’- CAG ATA TGC AGG GAG TCA CC-3’ |
|  | TNF-α | forward | 5’- ATC CGC GAC GTG GAA CTG-3’ |
|  |  | reverse | 5’- ACC GCC TGG AGT TCT GGA A -3’ |
|  | TGF-β | forward | 5’- TGG AGC AAC ATG TGG AAC TC-3’ |
|  |  | reverse | 5’- GTC AGC AGC CGG TTA CCA-3’ |
|  | IFN-γ | forward | 5’- CTG CTG ATG GGA GGA GAT GT-3’ |
|  |  | reverse | 5’- TTT GTC ATT CGG GTG TAG TCA-3’ |
|  | GAPDH | forward | 5’- CCA CTC CTC CAC CTT TGA C -3’ |
|  | ` | reverse | 5’- ACC CTG TTG CTG TAG CCA-3’ |
| Human | CXCL1 | forward | 5’- AACCGAAGTCATAGCCACA -3’ |
|  |  | reverse | 5’- TCCTAAGCGATGCTCAAA -3’ |
|  | CXCL2 | forward | 5’- CAAACCGAAGTCATAGCC -3’ |
|  |  | reverse | 5’- GAACAGCCACCAATAAGC -3’ |
|  | CXCL3 | forward | 5’- TGCTGCTCCTGCTCCTGGTG -3’ |
|  |  | reverse | 5’- AGTTGGTGCTCCCCTTGTTCA -3’ |
|  | CXCL5 | forward | 5’- GAGAGCTGCGTTGCGTTTGTTTAC -3’ |
|  |  | reverse | 5’- CCGTTCTTCAGGGAGGCTACCA -3’ |
|  | CXCL6 | forward | 5’- ATGACTTCCAAGCTGGCCGTGGCT -3’ |
|  |  | reverse | 5’- TCTCAGCCCTCTTCAAAAACTTCTC -3’ |
|  | CXCL7 | forward | 5’- GTAACAGTGCGAGACCACTTC -3’ |
|  |  | reverse | 5’- CTTTGCCTTTCGCCAAGTTTC -3’ |
|  | CXCL8 | forward | 5’- AACCGAAGTCATAGCCACA -3’ |
|  |  | reverse | 5’- TCCTAAGCGATGCTCAAA -3’ |
|  | MIF | forward | 5’- AGAACCGCTCCTACAGCAAG -3’ |
|  |  | reverse | 5’- GAGTTGTTCCAGCCCACATT -3’ |
|  | CXCR1 | forward | 5’- CCCTGCCCTTCTTCCTTTTC -3’ |
|  |  | reverse | 5’- ACACCATCCGCCATTTTGCT -3’ |
|  | CXCR2 | forward | 5’- CTCCAATAACAGCAGGTCAC -3’ |
|  |  | reverse | 5’- GGCTCAGCAGGAATACCA -3’ |
|  | E-cadherin | forward | 5’- ACA GCA CGT ACA CAG ACCC TA -3’ |
|  |  | reverse | 5’- GCA GAA GTG TCC CTG TCC CAG -3’ |
|  | N-cadherin | forward | 5’- TTG GAT CAA TGT CAT AAT CAA GTG CTG TA -3’ |
|  |  | reverse | 5’- CTC CTA TGA GTG GAA CAG GAA CG -3’ |
|  | Snail | forward | 5’- TGCAGGACTCTAATCCAAAGTTTACC -3’ |
|  |  | reverse | 5’- GAG GGA TGG CTG CCA GC -3’ |
|  | Vimentin | forward | 5’- AGC CGA AAA CAC CCT GCA AT -3’ |
|  |  | reverse | 5’- CGT TCA AGG GTC AAG ACG TGC -3’ |
|  | p16 | forward | 5’- TTCCTGGACACGCTGGT -3’ |
|  |  | reverse | 5’- CAATCGGGGATGTCTGAG -3’ |
|  | p21 | forward | 5’- GGATGTCCGTCAGAACCC -3’ |
|  |  | reverse | 5’- GCTCCCAGGCGAAGTCA -3’ |
|  | GAPDH | forward | 5’- AGGGCTGCTTTTAACTCTGGT -3’ |
|  | ` | reverse | 5’- CCCCACTTGATTTTGGAGGGA -3’ |
